# Soluble CD146, a biomarker and a target for preventing resistance to anti-angiogenic therapy in glioblastoma

**DOI:** 10.1186/s40478-022-01451-3

**Published:** 2022-10-23

**Authors:** Ahmad Joshkon, Emeline Tabouret, Wael Traboulsi, Richard Bachelier, Stéphanie Simoncini, Sandrine Roffino, Carine Jiguet-Jiglaire, Bassam Badran, Benjamin Guillet, Alexandrine Foucault-Bertaud, Aurelie S. Leroyer, Françoise Dignat-George, Olivier Chinot, Hussein Fayyad-Kazan, Nathalie Bardin, Marcel Blot-Chabaud

**Affiliations:** 1 Aix-Marseille University, INSERM1263, INRAE1260, C2VN, Marseille, France; 2 Aix-Marseille University, APHM, CNRS, INP, Service de Neuro-Oncologie CHU Timone, Marseille, France; 3 Department of Neuro-Oncology, Hôpitaux de Marseille, Marseille, France; 4 CNRS, ISM UMR 7287, Aix-Marseille University, Marseille, France; 5 Laboratory of Cancer Biology and Molecular Immunology, Faculty of Science, Lebanese University, Hadath, Lebanon; 6 Department of Radiopharmacy, La Timone University Hospital, CERIMED, Aix-Marseille University, Marseille, France

**Keywords:** Soluble CD146, Biomarker, Therapeutic antibody, Bevacizumab, Glioblastoma

## Abstract

**Rationale:**

Glioblastoma multiforme (GBM) is a primary brain tumor with poor prognosis. The U.S. food and drug administration approved the use of the anti-VEGF antibody bevacizumab in recurrent GBM. However, resistance to this treatment is frequent and fails to enhance the overall survival of patients. In this study, we aimed to identify novel mechanism(s) responsible for bevacizumab-resistance in CD146-positive glioblastoma.

**Methods:**

The study was performed using sera from GBM patients and human GBM cell lines in culture or xenografted in nude mice.

**Results:**

We found that an increase in sCD146 concentration in sera of GBM patients after the first cycle of bevacizumab treatment was significantly associated with poor progression free survival and shorter overall survival. Accordingly, in vitro treatment of CD146-positive glioblastoma cells with bevacizumab led to a high sCD146 secretion, inducing cell invasion. These effects were mediated through integrin αvβ3 and were blocked by mucizumab, a novel humanized anti-sCD146 antibody. In vivo, the combination of bevacizumab with mucizumab impeded CD146 + glioblastoma growth and reduced tumor cell dissemination to an extent significantly higher than that observed with bevacizumab alone.

**Conclusion:**

We propose sCD146 to be 1/ an early biomarker to predict and 2/ a potential target to prevent bevacizumab resistance in patients with glioblastoma.

**Supplementary Information:**

The online version contains supplementary material available at 10.1186/s40478-022-01451-3.

## Introduction

Glioblastoma multiforme (GBM) is the most common malignant primary brain tumor with extremely poor prognosis because of its diffusive and infiltrative nature. It accounts for more than 50% of all gliomas [1]. Despite decades of research, GBM remains among the most lethal of all forms of cancers. The five-year survival rate barely reaches 3% in patients and the median survival of patients is about 14 months, even after first line treatment including surgery, radiotherapy and chemotherapy. Resistance to this conventional line of treatment is systematic and results in tumor recurrence [2]. Vascular proliferation that is markedly powered by Vascular Endothelial Growth Factor (VEGF) signaling is a hallmark in GBM. Bevacizumab, a neutralizing monoclonal anti-VEGF antibody, has been recognized as a potent drug candidate in the treatment of glioblastoma at recurrence. Multiple phase III clinical trials including the AVAglio, RTOG 0825 and the EORTC 26,101 have confirmed a significantly longer progression-free survival (PFS) in GBM patients treated with bevacizumab. However, bevacizumab has no effect on overall survival. Indeed, bevacizumab was able to diminish tumor volume, intratumoral angiogenesis and oxygen consumption in vivo at early cycles of treatment, which could explain the increase in PFS, but why this did not translate into overall survival benefit remains to be investigated [3].

CD146 (MCAM) is a transmembrane glycoprotein belonging to the immunoglobulin superfamily [4]. CD146 is present on the whole vascular tree, mainly at the intercellular junction of endothelial cells and regulates cell–cell cohesion, permeability and angiogenesis. Besides, soluble CD146 (sCD146), generated by ectodomain shedding of membrane CD146, induces endothelial cell proliferation and enhances angiogenesis and metastasis [5]. In cancer, the expression of CD146 is associated with a more aggressive phenotype, malignant angiogenesis, thromboembolism, and resistance to certain chemotherapies. Alongside, sCD146 is regarded as a poor prognostic factor in various cancers [6]. Its plasma concentration is robustly increased in cancer patients as well as in the cerebrospinal fluid (CSF) of patients with central nervous system (CNS) diseases [7]. Public databases show that CD146 is expressed in more than 65% of patients with glioblastoma (Additional file 1: Fig. S1A) and that high expression of the molecule is associated with a reduced survival probability (Additional file 1: Fig. S1B). Of importance, CD146 acts as a coreceptor for VEGFR2, essentially potentiating VEGF angiogenic signaling [8]. In addition, CD146 has been recently shown to be overexpressed in renal cell carcinoma 786-O cells after acquiring resistance to the therapeutic agent Sunitinib, which blocks VEGFR2 signaling pathway [9]. Thus, we hypothesized that CD146 may be involved in the escape mechanism in recurrent GBM treated with Bevacizumab.

This study is thus aimed at deciphering a potential mechanism behind glioblastoma resistance to bevacizumab through the CD146/sCD146 signaling axis with the objective to block this escape pathway that hinders the in vivo effectiveness of bevacizumab.

## Materials and methods

### Cell culture

The three human glioblastoma cell lines U87, U118 and U373 were a kind gift from Dr. F. Peiretti (INSERM UMR-S 1263 Marseille, France). Cells were confirmed to be mycoplasma free. U118 and U373 cells were cultured in DMEM/F12 GlutaMAX™ supplemented with 10% heat inactivated FCS while U87 cells were cultured in DMEM (1 g/L glucose) enriched with 10% FCS. All media contained 100 U/ml penicillin, 100 µg/mL streptomycin and 2 mM L-glutamine. Cells were maintained in 5% CO2 at 37 °C. For the generation of bevacizumab resistant U87, U118 and U373, 2 × 10^5^ parental wild-type cells were cultured with 100 µg/mL of bevacizumab (Roche) in the culture media for up to 2 months. CHO cells were cultured in DMEM medium containing 10% FCS. Supplementary methods are given in the supplementary data file.

## Results

### Soluble CD146 is increased in the plasma of glioblastoma patients and its increase is associated with poor Progression-Free Survival (PFS) and Overall Survival (OS) after bevacizumab treatment

**Fig. 1:**
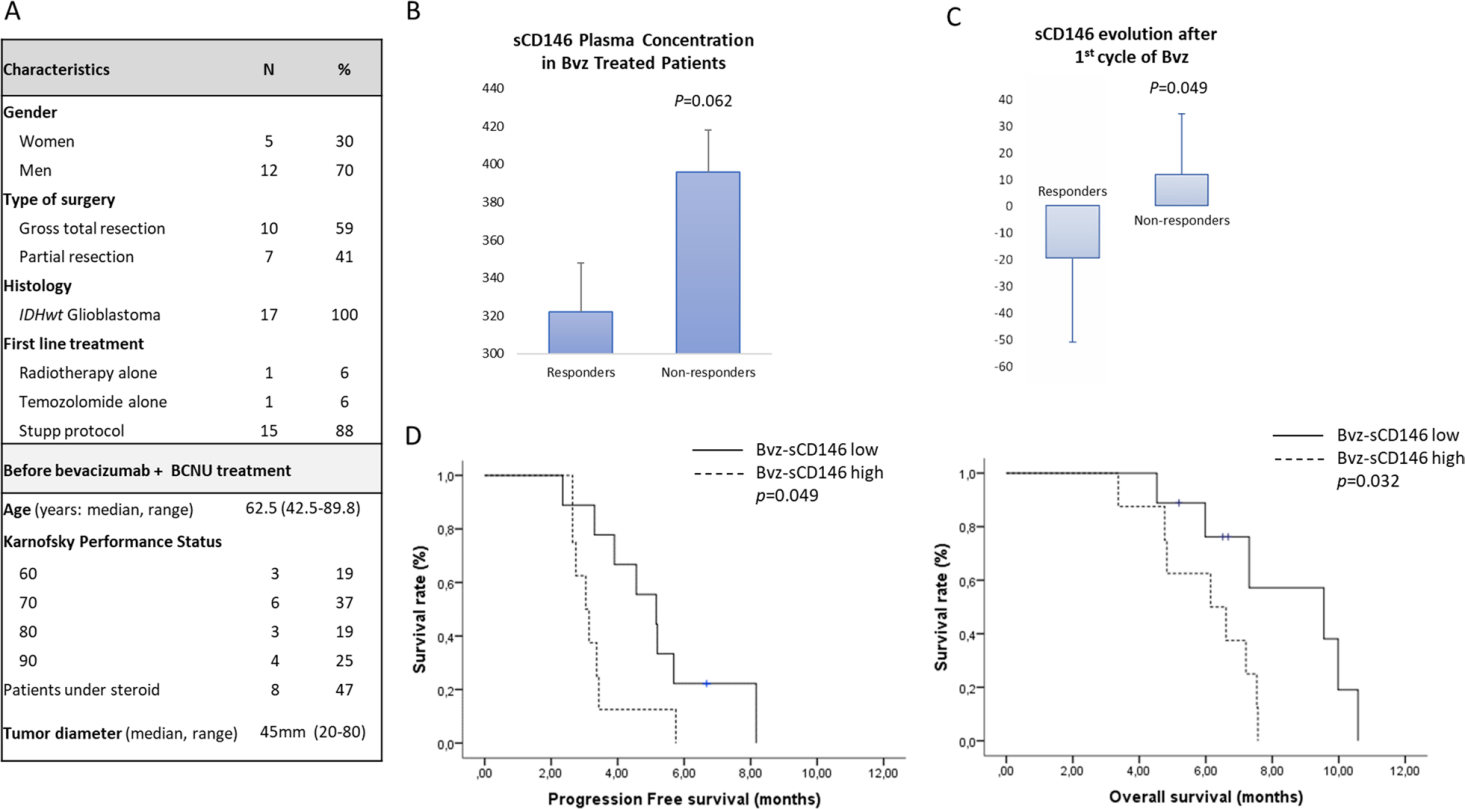
sCD146 plasma concentration in patients under bevacizumab treatment. **a** Patients’ characterization. **b** sCD146 plasma concentration in patients after one cycle of bevacizumab treatment (Bev) between responders and non-responders. **c** Evolution of sCD146 plasma concentration at baseline versus after one cycle of bevacizumab treatment, in responder and non-responder patients. **d** Progression-Free survival (left) and Overall survival (right) according to sCD146 plasma concentration in patients under bevacizumab treatment. *p < 0.05, non-responders versus responders

We measured the plasma concentration of soluble CD146 (sCD146) in a cohort of 17 patients with recurrent *IDHwt* glioblastoma who were treated for 3 weeks with bevacizumab and carmustine (Fig. 1a). Plasma samples were collected just before the second bevacizumab administration. Responses after bevacizumab-chemotherapy administration were either complete (1 patient) or partial (5 patients), corresponding to responders, or stable (4 patients) or progressive (7 patients), corresponding to non-responders. We show that sCD146 plasma level, under treatment, was higher in non-responder patients than in responders (Fig. 1b), and that this corresponded to an increase in sCD146 occurring after treatment (Fig. 1c). Importantly, a high sCD146 plasma level in patients treated with one cycle of bevacizumab significantly associated with shorter PFS (*p* = 0.049) and OS (*p* = 0.032) (Fig. 1d). Patients with a high sCD146 plasma concentration had a median PFS of 3.0 months (95% CI 2.5–3.6) as compared to 5.2 months (95% CI 3.4–6.9) in patients with low sCD146 plasma concentration. Patients with a high sCD146 plasma concentration had a median OS of 6.1 months (95% CI 3.7–8.6) versus 9.6 months (95% CI 4.8–14.3) in patients with low sCD146 plasma concentration. In contrast, neither age, Karnofsky score (KPS) nor steroid dose were associated with patient PFS or OS in univariate analyses (Additional file 1: Table S1), suggesting the independence of sCD146 from classical prognostic factors.

These results highlight the pejorative impact of sCD146 increase in patients treated with bevacizumab and its potential implication in bevacizumab resistance.

### Long-term treatment with Bevacizumab significantly induces CD146/soluble CD146 signaling pathway in CD146-positive glioblastoma cell lines

In this study, we used 3 different glioblastoma cells lines, U87, U373 and U118. Flow cytometry analysis of U87 and U373 glioblastoma cell lines demonstrated potent expression of CD146, VEGFR2 and integrin αvβ3 in these two cell lines. In contrast, U118 cells did not express integrin αvβ3 and VEGFR2, only faintly expressed CD146, and were thus used as a negative control (Additional file 1: Fig. S2). The secretion capacity of sCD146 and VEGF from the three glioblastoma cell lines was determined in vitro. ELISA experiments on culture supernatant evidenced a high secretion of sCD146 in U87 and U373 cell lines while U118 cells did not secrete sCD146. In contrast, U87, U373 and U118 highly secreted the Vascular Endothelial Growth Factor (VEGF) (Additional file 1: Fig. S3).

**Fig. 2:**
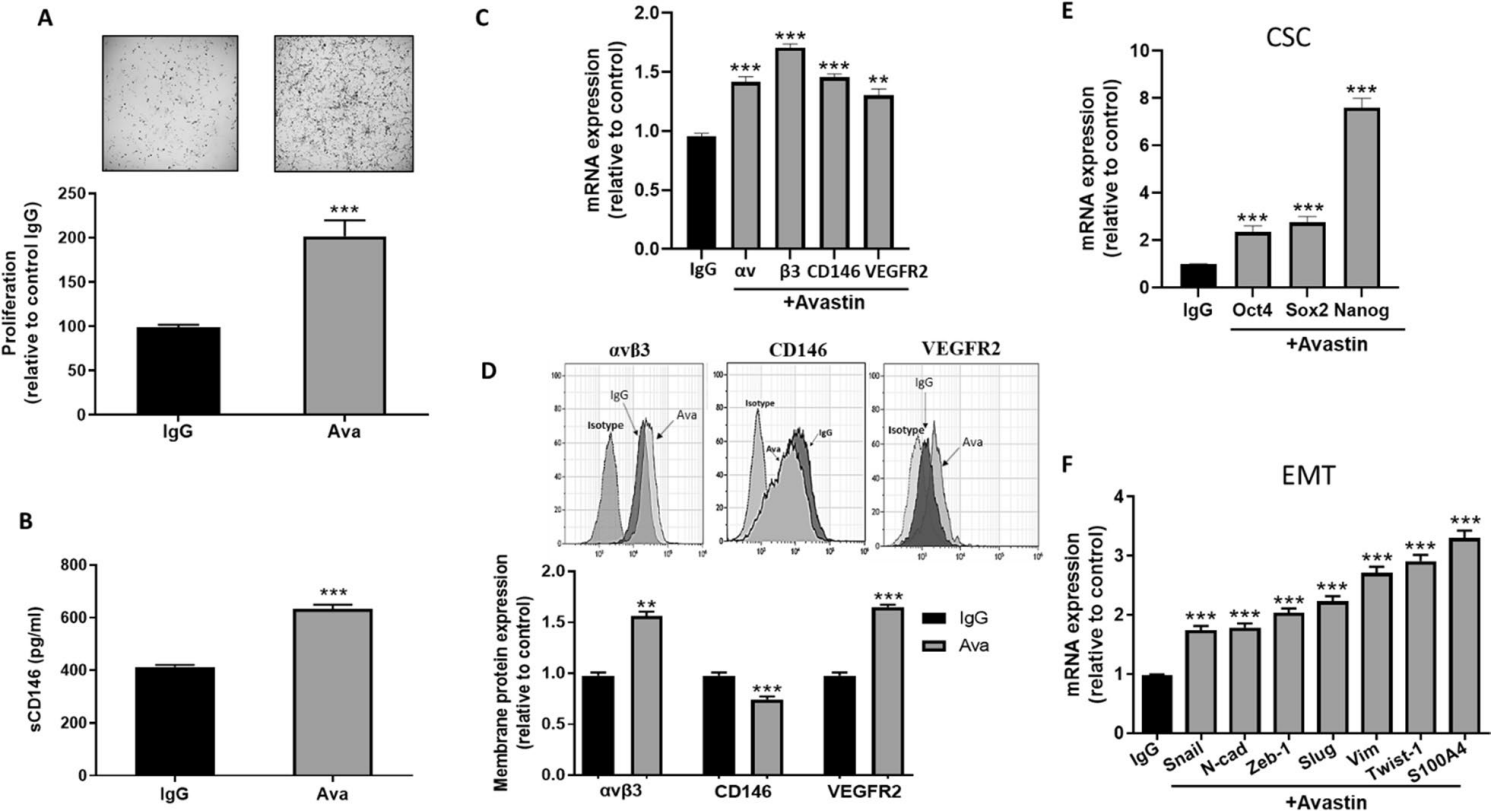
Bevacizumab induces proliferation and CSC/EMT markers in U87 cells. In-vitro challenging of U87 cells with bevacizumab (Avastin; 100 µg/ml) for 2 months enhanced cell proliferation (**a**) and sCD146 secretion (**b**), normalized to total cell number, as compared to IgG treated cells. Avastin-challenged U87 cells upregulated CD146, VEGFR2 and integrin subunits αv and β3 gene transcription (**c**) and their membrane expression, except for CD146 (**d**). U87 challenging with Avastin potently induced expression of markers related to CSC (**e**) and EMT (**f**) at the mRNA level as compared to control IgG treated cells. Average of 5 experiments is shown; **p < 0.01, ***p < 0.001, experimental vs control

To generate Bevacizumab-resistant GBM cell lines, we treated U87, U373 and U118 for two months with 100 µg/ml bevacizumab (commercially known as Avastin) or irrelevant IgG as a control. The absence of antibody toxicity was confirmed using trypan blue exclusion assay and live/dead fixable aqua staining dye (data not shown). After 2 weeks of treatment, we noticed a robust increase in U87 and U373 cell proliferation, but not in U118 cell proliferation, an effect that was maintained during the entire treatment course (Fig. 2a; Additional file 1: Figs. S4A; S5A). In addition, a significant increase in CD146, VEGFR2, integrin subunits αv and β3 at the mRNA levels was detected in U87 and U373 cell lines, but not in U118 cells (Fig. 2c; Additional file 1: Figs. S4C; S5C). As membrane CD146 expressed at the membrane can be shed to generate sCD146, we analyzed the membrane expression of these proteins by flow cytometry. The membarne expression of integrin αvβ3 and VEGFR2 were increased but CD146 membrane expression decreased (Fig. 2d; Additional file 1: Fig. S4D). In contrast, membrane expression of the three surface proteins did not change in U118 cells (Additional file 1: Fig. S5D).

Besides, sCD146 concentration was significantly increased in the culture media of U87 and U373 cells after exposure to bevacizumab as compared to irrelevant IgG (Fig. 2b; Additional file 1: Fig. S4B). This increase in sCD146 concentration was not detected in the supernatant of bevacizumab-treated U118 cells (Additional file 1: Fig. S5B).

We then examined Epithelial-Mesenchymal Transition (EMT) and Cancer Stem Cells (CSC) markers in the bevacizumab-challenged glioblastoma cells. We analyzed the main CSC markers oct4, sox2 and nanog, and the EMT markers that are commonly expressed in GBM cells, snail, slug, vimentin, N-cadherin, S100A4, Twist1, and Zeb1, at the mRNA level. All these markers were potently upregulated in the bevacizumab challenged U87 cell line as compared to IgG treated cells (Fig. 2e and f). Similar results were obtained in the U373 cell line (Additional file 1: Fig. S4 E–F) but not in the CD146-negative glioblastoma cell line U118 (Additional file 1: Fig. S5 E–F).

### Soluble CD146 reproduces the effects observed with long-term bevacizumab treatment in CD146-positive glioblastoma cells

**Fig. 3:**
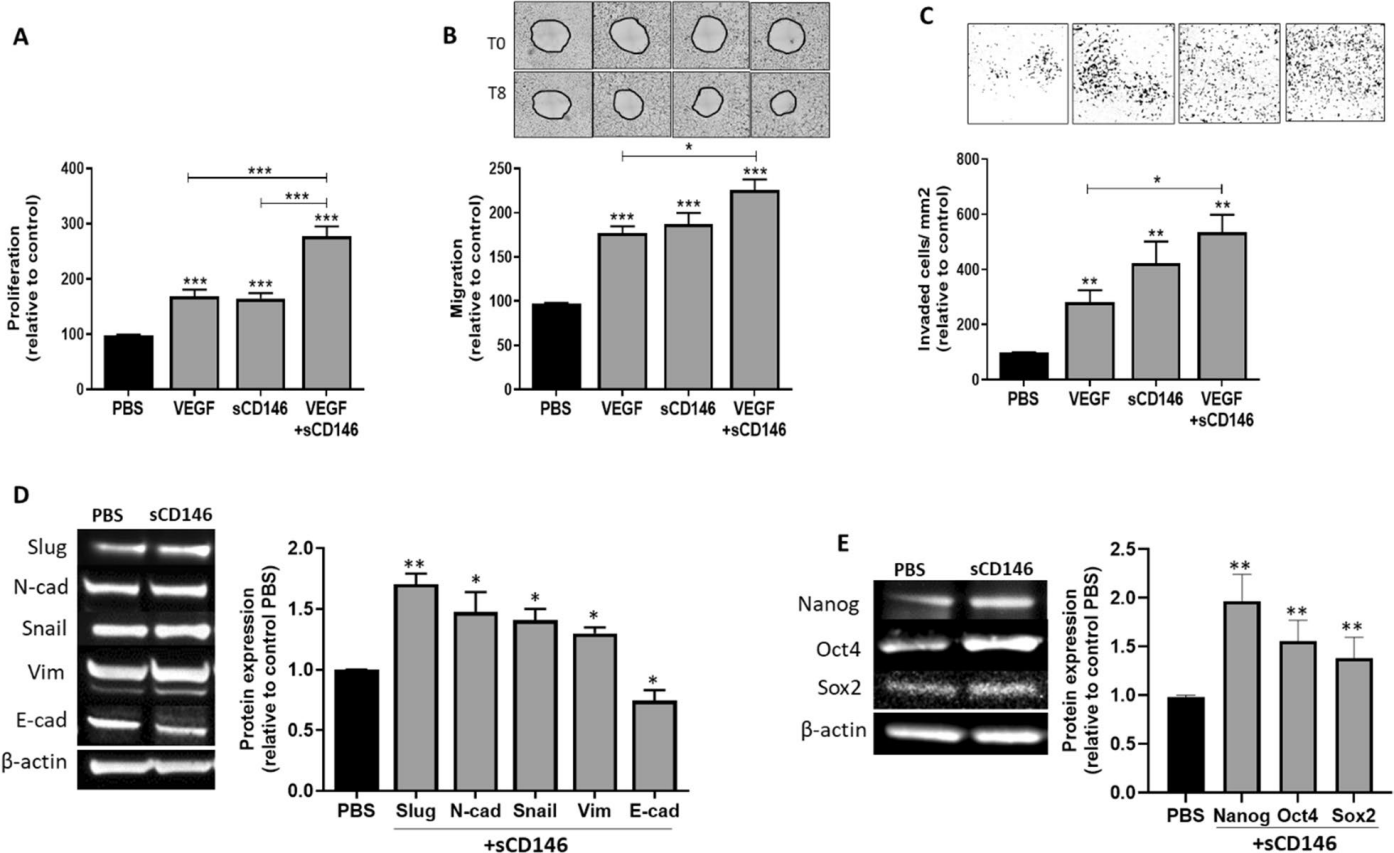
Soluble CD146 induces U87 cell proliferation, migration and invasion and promotes CSC/EMT markers. U87 cells were treated with 100 ng/ml of VEGF, rsCD146 or combination of both molecules for 48 h and cell proliferation (**a**), migration (**b**) and invasion (**c**) were determined. EMT (**d**) and CSC (**e**) markers were also examined after 48 h of treatment with 100 ng/ml sCD146. Representative blots from 5 experiments are shown. *p < 0.05, **p < 0.01, ***p < 0.001, experimental vs control

As bevacizumab treatment of CD146-positive (CD146 +) glioblastoma cells led to a potent increase in sCD146 secretion, we investigated sCD146 effect on U87 and U373 cells. Our results show that in vitro stimulation of these cells for 48 h with recombinant sCD146 or VEGF significantly increased cell proliferation, migration, and invasion. An additive outcome effect was achieved when combining both molecules, in comparison to VEGF or sCD146 treatment alone (Fig. 3a–c; Additional file 1: Fig. S6 A–C). In contrast, in U118 cells, sCD146 or VEGF failed to induce cell proliferation (Additional file 1: Fig. S7).

Moreover, sCD146 potently induced the expression of the mesenchymal markers snail, slug, N-cadherin and vimentin, but decreased the epithelial marker E-cadherin at the protein level in U87 cells. Likewise, sCD146 upregulated the expression of the cancer stem cell markers nanog, oct4 and sox2 in U87 cells (Fig. 3d and e). Similar results were obtained in U373 cell lines (Additional file 1: Fig. S6 D and E).

### Soluble CD146 mediates its effects through integrin αvβ3 in CD146-positive glioblastoma cells

**Fig. 4:**
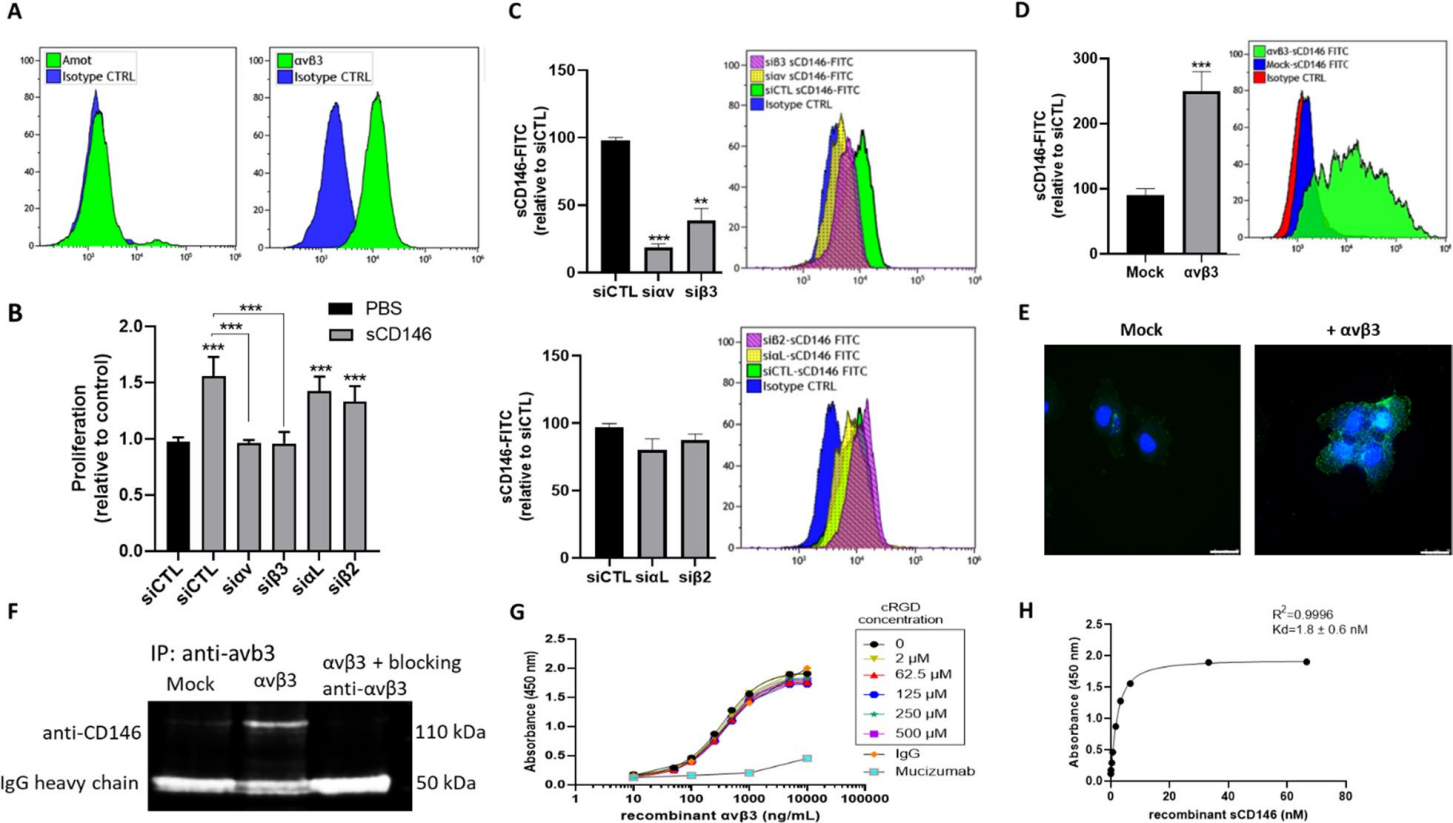
Soluble CD146 binds integrin αvβ3 on U87 cells. The expression of angiomotin (Amot) and αvβ3 on U87 cells was analyzed by flow cytometry (**a**). The effect of silencing RNA targeting αv, β3, αL and β2 was analyzed on sCD146-induced U87 cell proliferation (**b**). U87 cells were transfected with siRNA targeting αv, β3, αL and β2 and sCD146-FITC binding was determined by flow cytometry (**c**). CHO cells were co-transfected with plasmids encoding integrin subunits αv and β3 or control plasmids (mock) and sCD146-FITC binding was determined by flow cytometry (**d**) or by immunofluorescence microscopy (**e**). Mock transfected or αvβ3 expressing CHO cells were treated or not with blocking anti-αvβ3 antibody and then sCD146 was added for 1 h at 37°. CD146 was examined after immunoprecipitation with anti- αvβ3 antibody. Loading was analyzed using IgG heavy chain (**f**). In two ELISA assays, the effect of cyclic RGD peptide/mucizumab (**g**) and the dissociation constant Kd (**h**) were estimated for the binding of αvβ3 to rsCD146. White bars correspond to 25 µm. Average of 3 experiments is shown; *p < 0.05, **p < 0.01, ***p < 0.001, experimental vs control

In a previous work, we showed that sCD146 interacts with angiomotin on endothelial cells to promote angiogenesis [10]. Thus, we examined angiomotin expression on U87, U373, and U118 cells. Flow cytometry analysis showed a weak expression of this surface protein (Fig. 4a; Additional file 1: Fig. S2). In contrast, integrin subunits αv and β3 were expressed by U87 and U373 but not U118 cells as assessed by RT-PCR and flow cytometry. Therefore, we tested a possible interaction between sCD146 and integrin αvβ3. Silencing RNA targeting integrin subunits αv or β3 significantly reduced sCD146-induced increase in proliferation of U87 and U373 cells, whereas siRNA targeting other integrin subunits, αL or β2, did not modify cell proliferation (Fig. 4b; Additional file 1: Fig. S8A). Moreover, knocking down integrin αvβ3 using silencing RNA targeting either αv or β3 subunits, but not αL or β2 integrins, on U87 and U373 cells, led to a significant decrease in sCD146-FITC binding to these cells (Fig. 4c; Additional file 1: Fig. S8B). In order to confirm the specific binding of sCD146 on αvβ3, we transfected Chinese Hamster Ovary cells (CHO), which originally do not express this integrin, with αvβ3. Exogenous expression of integrin subunits αv and β3 into CHO cells induced a significant increase in sCD146-FITC binding to these cells as revealed by flow cytometry and immunofluorescence experiments (Fig. 4d and e). In addition, anti-αvβ3 antibody immunoprecipitated sCD146 only when CHO cells expressed this integrin after sCD146 treatment. To confirm the specificity of the immunoprecipitated sCD146, we treated CHO cells with blocking anti-αvβ3 antibody before adding sCD146. Results show that anti-αvβ3 antibody potently reduced the interaction with sCD146 (Fig. 4f).

In-house ELISA also confirmed a strong interaction between integrin αvβ3 and sCD146 with an estimated dissociation constant of 1.8 nM. The addition of blocking cyclic RGD peptide failed to inhibit this interaction even at high concentrations, in contrast to a newly generated humanized neutralizing monoclonal anti-sCD146 antibody, mucizumab, which was generated by our laboratory (Fig. 4g and h).

Finally, we overexpressed or silenced integrin αvβ3 in U118 and U87 cells and studied EMT markers in response to sCD146 stimulation. Results showed that only cells expressing αvβ3 upregulate EMT markers when treated with sCD146 (Additional file 1: Figs. S9; S10).

### CD146 is part of a signalosome containing VEGFR2 and Integrin αvβ3

To analyze the components of the signalosome containing CD146, we performed co-immunoprecipitation experiments using anti-CD146 antibody, S-Endo1. Immunoblotting with anti-VEGFR2, anti-αv, and anti-β3 antibodies revealed specific bands corresponding to these proteins. Membrane probing with an antibody to another surface protein, EPCAM, failed to detect it, attesting the specificity of the immunoprecipitated proteins (Additional file 1: Fig. S11 A). Results were reproduced in U373 cell line (Additional file 1: Fig. S12 A).

As CD146 is a co-receptor for VEGFR2, and since VEGFR2 activation induces its own phosphorylation at multiple tyrosine residues [11], we investigated whether sCD146 or VEGF induces the phosphorylation of CD146 and whether integrin αvβ3 is indispensable in this process. Accordingly, U87 cells were treated with either sCD146 or VEGF, and CD146 was immunoprecipitated followed by western blotting using anti-pan phospho-tyrosine antibody. Results showed that sCD146 and VEGF increased the phosphorylation of membrane CD146 (Additional file 1: Fig. S11 B). Next, we knocked-down integrin αvβ3 using silencing RNA and reproduced the experiment. A potent decrease in CD146 phosphorylation in response to sCD146 or VEGF stimulation was detected as compared to control cells transfected with scrambled RNA (Additional file 1: Fig. S11 B). These results were reproduced in U373 cells (Additional file 1: Fig. S12 B).

We then studied the phosphorylation of integrin β3. Our results show a significant increase in integrin β3 phosphorylation in response to sCD146 or VEGF stimulation (Additional file 1: Fig. S11 C). To examine if CD146, integrin αvβ3, or VEGFR2 are crucial in the phosphorylation and activation of the newly defined signalosome, we used CRISPR-Cas9 technology to individually knockout each of these proteins (Additional file 1: Fig. S13). We showed that the knockout of CD146 on U87 cells significantly reduced integrin β3 and VEGFR2 phosphorylation following sCD146 or VEGF stimulation. Also, knocking out integrin β3 on U87 cells significantly reduced or abolished VEGFR2 phosphorylation in response to VEGF or sCD146 stimulation (Additional file 1: Fig. S11 D). In contrast, VEGFR2 knockout on U87 cells abrogated integrin β3 phosphorylation in response to VEGF stimulation but not sCD146 (Additional file 1: Fig. S11 C). Similar results were obtained in U373 cells (Additional file 1: Fig. S12 C-D).

Of interest**,** we also showed this signalosome to exist in human umbilical endothelial cells, HUVEC, and revealed that sCD146, through integrin αvβ3, induces cell proliferation and migration (Additional file 1: Fig. S14).

These results demonstrate a complex constituted by the surface proteins CD146, VEGFR2 and integrin αvβ3 that mediates sCD146 and VEGF effects on CD146 + glioblastoma cells, but also on endothelial cells.

### Soluble CD146 and VEGF activate common signaling pathways in CD146-positive glioblastoma cell lines

We next investigated the signaling pathways that are implicated in mediating sCD146 and VEGF effects in CD146 + glioblastoma cells. To this end, we studied the phosphorylation of key proteins involved in major signaling pathways regulating cell survival, proliferation, and invasion. Our results show that, similar to VEGF, sCD146 induced the phosphorylation of P38 MAPK, AKT, and ERK44/42 proteins. Besides, both sCD146 and VEGF induced the phosphorylation and activation of focal adhesion kinase (FAK). The phosphorylation of these proteins became significantly impaired when either CD146, VEGFR2, or integrin αvβ3 were knocked out (Additional file 1: Figs. S15 and S16). These effects were functionally translated to a decrease in the ability of cells to proliferate and migrate in response to sCD146 and/ or VEGF stimulation (Additional file 1: Figs. S17 and S18).

Thus, CD146, VEGFR2 and integrin αvβ3 interact together to translate extracellular stimuli into intracellular signals, and each of these proteins is required to potentiate the activated signaling pathways.

### A novel humanized anti-sCD146 antibody, mucizumab, blocks sCD146 adverse effects on CD146-positive glioblastoma cells and prevents escape from bevacizumab in vitro

**Fig. 5:**
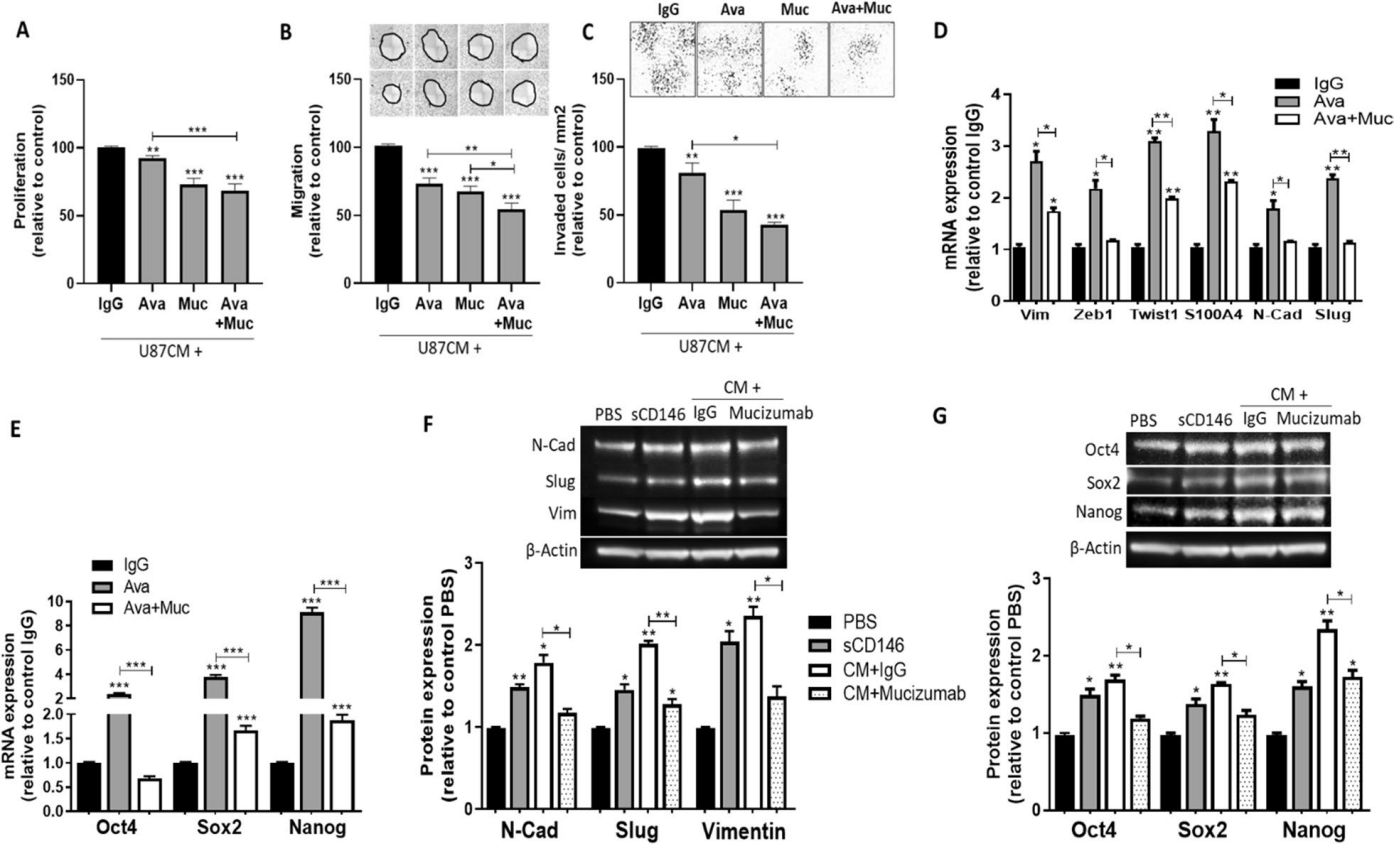
Humanized anti-sCD146 antibody mucizumab significantly decreases U87 cell proliferation, migration and invasion and hampers CSC and EMT in vitro. U87 cells were treated with conditioned media (CM) containing Irrelevant IgG, bevacizumab (Avastin), Mucizumab, or combination of both antibodies for proliferation (**a**), migration (**b**), and invasion assays (**c**). EMT and CSC markers were also examined at the mRNA (**d** and **e**) and protein (**f** and **g**) levels. Average of 3 experiments is shown; *p < 0.05, **p < 0.01, ***p < 0.001, experimental vs control

In order to block glioblastoma escape from bevacizumab, we tested the effect of the humanized neutralizing monoclonal anti-sCD146 antibody, mucizumab. The addition of 100 µg/ml of either bevacizumab, mucizumab, or the combination of both antibodies to U87 or U373 conditioned media (CM) resulted in a significant decrease in cell proliferation, migration, and invasion in vitro. Our results showed that mucizumab displayed greater effects than that achieved by bevacizumab, while the combination of mucizumab and bevacizumab significantly extended the inhibitory effects of bevacizumab on cells’ proliferation, migration, and invasion as compared to bevacizumab treatment alone (Fig. 5a–c; Additional file 1: Fig. S19 A-C).

We then reproduced the long-term bevacizumab challenging experiment and added mucizumab to the media along with bevacizumab. The effect on EMT and CSC induction in U87 and U373 cells was evaluated. Our results showed a robust decrease in the tested EMT and CSC markers when mucizumab was added to bevacizumab (Fig. 5d–g; Additional file 1: Fig. S19 D-E).

These data confirm that CD146 + glioblastoma cells escape bevacizumab through sCD146 secretion.

### Combination of bevacizumab with mucizumab exhibits greater inhibitory effects than bevacizumab alone on tumor growth in different preclinical models

**Fig. 6:**
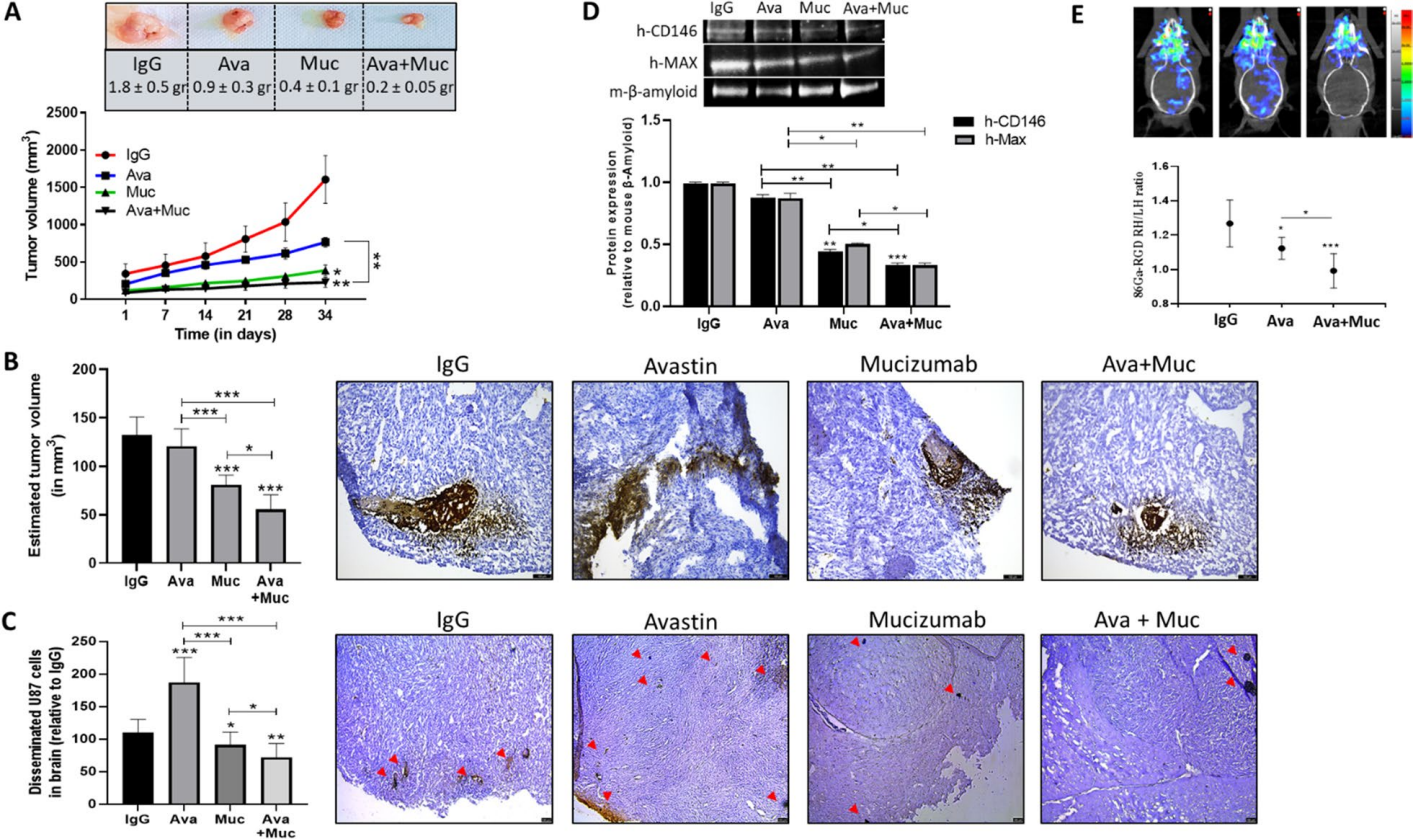
Humanized anti-sCD146 antibody, mucizumab, significantly reduces tumor growth in two different in-vivo models and displays complementary effects with bevacizumab. Tumor volume was measured in 20 athymic nude mice subcutaneously injected with U87 cells after treatment with IgG, bevacizumab (Avastin), mucizumab or bevacizumab + mucizumab. 5 mice were used in each group. Representative images of tumors and their weight are shown (**a**). 40 mice were orthotopically injected with U87 cells and treated with IgG, Avastin, mucizumab or bevacizumab + mucizumab. 10 mice were used in each group. Tumor volume was estimated based on immunohistochemical staining of human CD146 in serially cut mice brain sections (**b**). Tumor cell dissemination across all the brain was also determined. Red arrows show disseminated cells (**c**). Expression of human specific CD146 and MAX proteins in total brain lysate was determined in each group (**d**). In each group, imaging was performed in 6 representative mice orthotopically bearing U87 tumors using PET-scan after 86 Ga-RGD injection. Results were expressed as ratio of radioactivity between right (RH) and left (LH) hemispheres (**e**). Representative images are shown. *p < 0.05, **p < 0.01, ***p < 0.001, experimental vs control

To investigate the in vivo relevance of combining bevacizumab with mucizumab, U87 cells were xenografted subcutaneously in athymic nude mice and tumor growth was weekly monitored with caliper. After 5 weeks of treatment, results showed that mice receiving mucizumab developed significant smaller tumors than those receiving IgG. Of importance, an additive inhibitory effect on tumor growth was detected when bevacizumab was combined with mucizumab (Fig. 6a). We measured the concentrations of human VEGF (hVEGF) and human sCD146 (hsCD146) in the plasma of treated mice. Our results showed that the combination of bevacizumab and mucizumab significantly decreased hVEGF and hsCD146 concentrations in the plasma from treated mice as compared to IgG-control group whereas bevacizumab alone increased hsCD146 (Additional file 1: Fig. S20 A-B).

We also performed another in vivo study by orthotopically injecting U87 cells in nude mice. To evaluate the therapeutic benefit of the administered antibodies, immunohistochemistry was done to estimate tumor growth. Similar to that in ectopic tumor model, mucizumab significantly reduced tumor volume and metastasis, while the combination of mucizumab and bevacizumab greatly enhanced these effects. In contrast, bevacizumab, as a monotherapy, did not decrease intracranial tumor growth but rather increased tumor dissemination across the brain parenchyma (Fig. 6b and c). In addition, immunoblots using brain lysate from treated mice showed that the human proteins CD146 and MAX (a DNA-binding transcriptional regulator used as a human protein internal control) decreased when treating with bevacizumab and mucizumab to reach barely detectable levels when both antibodies were combined as compared to IgG-treated group (Fig. 6d). This indicates a large decrease in human tissue corresponding to GBM after treatment with both antibodies. This was confirmed by PET imaging showing a slight significant decrease of the tumor when bevacizumab was used, whereas a large decrease was observed in the presence of both bevacizumab and mucizumab. Along this line, the decrease was significantly higher in the bevacizumab + mucizumab group, as compared to bevacizumab group (Fig. 6e). Also, we showed a significant decrease in plasma concentration of hVEGF and hsCD146 in mice receiving simultaneously bevacizumab and mucizumab antibodies as compared to IgG-control group (Additional file 1: Fig. S20 C-D).

These results emphasize the interest of combining anti-VEGF and anti-sCD146 therapy for achieving maximal inhibitory effects on glioblastoma growth.

## Discussion

Currently, no curative treatment is available for glioblastoma. Despite new therapeutic options, including bevacizumab, it remains a disease with poor prognosis. Indeed, bevacizumab treatment is often accompanied with escape mechanisms leading to pejorative evolution. In this study we show that, in patients with glioblastoma treated with bevacizumab, an increase in sCD146, an easily quantifiable circulating biomarker, as soon as at the end of the first cycle of treatment is associated with a decrease in PFS and OS. Thus, sCD146 could constitute an early marker of resistance to this therapy. To confirm this hypothesis, we deciphered the sCD146-mediated molecular mechanisms leading to bevacizumab escape. We show in vitro that bevacizumab-resistant CD146 + glioblastoma cells are able to potently increase CD146 expression and enhance sCD146 secretion. The secreted sCD146 is able to exert autocrine effects on the glioblastoma cells to stimulate their proliferation, migration, and invasion. In the same way, sCD146 robustly increases cellular markers of epithelial to mesenchymal transition and cancer stem cell markers in CD146 + glioblastoma cells. These autocrine effects are mediated through the interaction of sCD146 with integrin αvβ3 which is overexpressed by glioblastoma cells after bevacizumab treatment. To block sCD146 adverse effects, we generated a novel humanized monoclonal anti-sCD146 antibody, mucizumab, that specifically targets and neutralizes the soluble form of CD146 while maintaining minimal reactivity to membrane CD146 which displays physiological functions. Of importance, the combination of bevacizumab and mucizumab antibodies exhibits a potential therapeutic benefit by significantly decreasing EMT and CSC in CD146 + bevacizumab-resistant glioblastoma cells in vitro, as well as potently decreasing tumor growth in a pre-clinical model of glioblastoma orthotopically injected in nude mice.

It is well established that epithelial to mesenchymal transition (EMT) and cancer stem cells (CSC) generation are critical for producing highly invasive and therapeutic-resistant cancer cells [12]. Cancer cells, especially GBM, are known to secrete soluble factors that can exert autocrine effects to drive tumor progression through EMT and CSC generation [13]. Here we show that the expression of CD146, integrin subunits αv and β3, and VEGFR2 are upregulated in response to bevacizumab treatment in CD146 + glioblastoma cells, and that they secrete high levels of sCD146. Despite the increase in CD146 at the mRNA level, the surface expression of this protein decreased while integrin αvβ3 and VEGFR2 increased. This may correspond to an increase in the shedding of membrane CD146 to generate the soluble form. Soluble CD146, in turn, induces a potent increase in EMT and CSC markers, suggesting an autocrine effect on glioblastoma cells. Recently, the angiogenic growth factor VEGF was shown to exert autocrine effect on glioblastoma cells to promote their invasion [14]. By comparing with VEGF, we show sCD146 to potently induce glioblastoma cell proliferation, migration, and invasion to an extent greater than that induced by VEGF. Of importance, the combination of both soluble factors exhibits an additive effect.

A huge body of evidence highlights the role of sCD146 in neoangiogenesis and tumor development. Despite these findings, it is unclear how sCD146 mediates its effect on target cells and most of its receptors remain to be discovered. We previously showed sCD146 to interact with angiomotin (Amot), an angiostatin binding protein, on endothelial cells [10]. Amot is also present on different cancer cells [15, 16]. Surprisingly, we found Amot to be absent or only weakly expressed on the three glioblastoma cell lines, leading to hypothesize that another receptor could mediate sCD146 effects. As angiostatin is known to bind Amot and integrin αvβ3 [17], and as increasing data emphasize the role of integrin αvβ3 in the process of neoangiogenesis [18], we hypothesized that integrin αvβ3 could constitute another sCD146 receptor. Through a series of experiments, we showed sCD146 to specifically interact with integrin αvβ3. We estimated the dissociation constant (K_d_) to be around 1.8 nM for sCD146. This is in line with the article of Wang et al. showing sCD146 to interact also with integrin αvβ1 on endothelial cells where it regulates the blood–brain barrier (BBB) permeability [7]. Of interest, the fact that the interaction between sCD146 and integrin αvβ3 was independent of RGD peptide, as revealed by competitive ELISA assay, suggested that sCD146 and cRGD have different binding sites on αvβ3.

A recent article by Jiang T et al. showed that CD146 acts as a coreceptor for VEGFR2 [8]. Besides, Masson-Gadais et al. showed that integrin αvβ3 is indispensable for the full activation and phosphorylation of VEGFR2 in response to VEGF stimulation on endothelial cells [19]. We thus investigated the activated signaling pathways after the interaction between sCD146 and integrin αvβ3. Both sCD146 and VEGF activated the same signaling pathways as they significantly increased ERK44-42, MAPK P38, Akt, and FAK phosphorylation. When CD146, integrin β3 or VEGFR2 were knocked-out, the activation of the signaling pathways as induced by sCD146 or VEGF were hampered. In addition, knocking out any of these proteins resulted in constitutive phosphorylation and activation of the ERK42-44, MAPK P38, Akt, and FAK proteins at the basal level. These results are in line with those of Szabo E et al. showing that VEGFR2 silencing on glioblastoma cell lines LN-308, LN-428 and T-325 potently increased the phosphorylation of AkT, ERK44/42, and P38 MAPK [20]. It appears that the absence of VEGFR2 or any of its co-receptors could trigger a stress response by CD146 + glioblastoma cells, which will compensate by constitutively activating alternative receptors such as CD146 or integrin αvβ3 and the subsequent signaling pathways. Based on these data and in view of our findings, we propose a signalosome composed of membrane CD146, VEGFR2 and integrin αvβ3 on glioblastoma cells that mediates sCD146 effects and regulates the MAPK/FAK signaling axis which will, in turn, control cell proliferation, migration and invasion. Of interest, co-immunoprecipitation experiments confirmed this signalosome to exist also on endothelial cells which highlights its importance in both tumor development and neoangiogenesis.

In this study, we show for the first time that sCD146, as VEGF, induces the phosphorylation of integrin β3 which consequently activates and phosphorylates FAK. The phosphorylation of integrin β3 was abolished when CD146 was knocked out and was significantly reduced in VEGFR2 knock out cells. Together, these data suggest that the interaction between CD146 and integrin αvβ3 induces conformation changes in integrin αvβ3 to enhance its affinity for sCD146. Concomitantly, VEGFR2 and CD146 phosphorylation could lead to the recruitment of adaptor proteins that bring tyrosine kinases close to the β subunit of αvβ3 integrin. In addition, we show sCD146 and VEGF to significantly induce CD146 phosphorylation on tyrosine residue Y641, an effect dependent on integrin αvβ3. This suggests that the binding of sCD146 or VEGF to their cognate receptors induces the phosphorylation of integrin αvβ3 which in turn brings protein kinases close to CD146. In accordance with these data, Xu W et al. showed that binding of growth factors to receptor tyrosine kinases (RTKs) induces CD146 phosphorylation which allows to recruit and interact with mTORC2 to induce endothelial cell proliferation. In the absence of CD146, VEGF proliferative effect on ECs vanished [21]. This further strengthens our findings showing that the binding of VEGF to VEGFR2 activates integrin αvβ3 which increase CD146 phosphorylation and eventually potentiate VEGF downstream signaling pathways.

Finally, we found sCD146, as VEGF, to strongly induce VEGFR2 phosphorylation. This effect was abolished when either CD146 or integrin β3 were knocked out. This signifies that sCD146 could interact with integrin αvβ3, phosphorylate integrin β3 which, in turn, facilitate CD146 phosphorylation to subsequently activate and phosphorylate VEGFR2 (Additional file 1: Fig. S21). It also emphasizes the significant role of integrin αvβ3 in enhancing VEGF effect on target cells. In fact, Cilengitide, a cyclic RGD pentapeptide inhibitor of αv integrins, failed to pass phase III clinical trials and showed no survival benefit for glioblastoma patients [22]. Interestingly, we found that cRGD and sCD146 have probably different binding sites on αvβ3, explaining, at least in part, why Cilengitide and other inhibitors of RGD binding integrins fail to block angiogenesis and tumor progression in several clinical trials.

The adverse effects of sCD146 thus appear to be more and more prominent in cancers and especially in glioblastoma [23]. To combat these undesirable effects, we developed a fully humanized monoclonal anti-sCD146 antibody, mucizumab. Mucizumab significantly reduces U87 and U373 cell proliferation, migration and invasion in vitro. Notably, combining mucizumab to bevacizumab induces an additive inhibitory effect on cancer cell proliferation, migration and invasion when compared to bevacizumab treatment as a monotherapy. Likewise, in the bevacizumab resistant cells, addition of mucizumab to the media significantly reduces the expression of EMT and CSC markers. These results were expanded and confirmed in vivo in two different U87 mouse models with ectopic and orthotopic injection of glioblastoma cells. In both cases, mucizumab was able to significantly reduce tumor growth in vivo. Importantly, higher inhibitory effect on tumor growth was observed when mucizumab and bevacizumab were combined. Of remark, bevacizumab monotherapy failed to significantly diminish tumor development in vivo, in agreement with the outcome of several phase III clinical trials [24]. By quantifying human VEGF and human sCD146 originating from the human tumor cells in the plasma of mice, we observed that bevacizumab reduced hVEGF concentration but increased hsCD146. However, combining bevacizumab with mucizumab led to a potent decrease in both hVEGF and hsCD146.

## Conclusion

This study identifies sCD146 as 1/ an easily detectable and early non-invasive biomarker to predict and 2/ a potential target to prevent bevacizumab resistance in patients with glioblastoma. This leads us to propose that a targeted therapy combining bevacizumab and mucizumab may enhance therapeutic benefits in patients with CD146 + glioblastoma.

## Supplementary Information

**Additional file 1.** **Supplementary methods:**Peptides and antibodies. Plasmids, siRNA and cell transfection. Western-blot and Immunoprecipitation assays. ELISA experiments. Immunofluorescence experiments. Flow cytometry experiments. Reverse Transcription-quantitative PCR (RT-qPCR). Cell migration assays. Cell Proliferation assays. Transwell invasion assays. Crispr/Cas9 deletion of genes. Experiments on animals and imaging. Immunohistochemistry. Patient cohort. Statistical analysis. **Supplementary figures and tables:** Supplementary Table 1: Absence of significant impact of age, KPS and steroid dose on progression-free survival or overall survival. Supplementary Table 2: references and application condition of the different antibodies used in the study. Supplementary Figure 1: Expression of CD146 on various types of cancers and effect on overall survival in patients with GBM. Supplementary Figure 2: Glioblastoma cell lines characterization. Supplementary Figure 3: Concentration of VEGF and sCD146 in the culture media of U87, U373, and U118 glioblastoma cell lines. Supplementary Figure 4: Avastin induces proliferation, sCD146 secretion, and EMT/CSC markers in U373 cells. Supplementary Figure 5: Avastin has no effect on CD146-negative U118 glioblastoma cells. Supplementary Figure 6: sCD146 induces U373 cell proliferation, migration and invasion in-vitro and promotes CSC and EMT markers. Supplementary Figure 7: Effect of sCD146 and VEGF on CD146-negative glioblastoma cells. Supplementary Figure 8: sCD146 binds integrin αvβ3 on U373 cells. Supplementary Figure 9: Soluble CD146 induces EMT in U118 cells transfected with integrin αvβ3. Supplementary Figure 10: Knocking-down integrin αvβ3 inhibits sCD146-induced EMT in U87 cells. Supplementary Figure 11: sCD146 mediated its effects on U87 cells through a signalosome containing CD146, αvβ3, and VEGFR2. Supplementary Figure 12: sCD146 mediates its effects on U373 cells through a signalosome containing CD146, αvβ3, and VEGFR2. Supplementary Figure 13: Validating gene knock out in U87 and U373 cells. Supplementary Figure 14: Integrin αvβ3 associates with membrane CD146 and VEGFR2 on HUVECs and binds sCD146. Supplementary Figure 15: CD146/VEGFR2/integrin αvβ3 signalosome mediates sCD146 and VEGF effects in U87 cells and activates common signaling pathways. Supplementary Figure 16: CD146/VEGFR2/integrin αvβ3 signalosome mediates sCD146 and VEGF effects in U373 cells and activates common signaling pathways. Supplementary Figure 17: CD146, integrin αvβ3, and VEGFR2 are essential for mediating sCD146 and VEGF effects on U87 cells. Supplementary Figure 18: CD146, integrin αvβ3, and VEGFR2 are essential for mediating sCD146 and VEGF effects on U373 cells. Supplementary Figure 19: Humanized anti-sCD146 antibody mucizumab significantly decreases U373 cell proliferation, migration and invasion and hampers CSC and EMT in-vitro. Supplementary Figure 20: Humanized anti-sCD146 mucizumab significantly decreases human sCD146 and human VEGF in two different mouse models of glioblastoma. Supplementary Figure 21: Illustrative summary describing the mechanism of resistance to bevacizumab in CD146-positive glioblastoma cells.
