# Supplementary material for: Soluble CD146, a biomarker and a target for preventing resistance to anti-angiogenic therapy in glioblastoma
Source: Acta Neuropathol Commun. 2022 Oct 23;10:151. doi: 10.1186/s40478-022-01451-3 (PMC9590138; doi:10.1186/s40478-022-01451-3)
Supplement: Supplementary file 1 — Additional file 1. Supplementary methods:Peptides and antibodies. Plasmids, siRNA and cell transfection. Western-blot and Immunoprecipitation assays. ELISA experiments. Immunofluorescence experiments. Flow cytometry experiments. Reverse Transcription-quantitative PCR (RT-qPCR). Cell migration assays. Cell Proliferation assays. Transwell invasion assays. Crispr/Cas9 deletion of genes. Experiments on animals and imaging. Immunohistochemistry. Patient cohort. Statistical analysis. Supplementary figures and tables: Supplementary Table 1: Absence of significant impact of age, KPS and steroid dose on progression-free survival or overall survival. Supplementary Table 2: references and application condition of the different antibodies used in the study. Supplementary Figure 1: Expression of CD146 on various types of cancers and effect on overall survival in patients with GBM. Supplementary Figure 2: Glioblastoma cell lines characterization. Supplementary Figure 3: Concentration of VEGF and sCD146 in the culture media of U87, U373, and U118 glioblastoma cell lines. Supplementary Figure 4: Avastin induces proliferation, sCD146 secretion, and EMT/CSC markers in U373 cells. Supplementary Figure 5: Avastin has no effect on CD146-negative U118 glioblastoma cells. Supplementary Figure 6: sCD146 induces U373 cell proliferation, migration and invasion in-vitro and promotes CSC and EMT markers. Supplementary Figure 7: Effect of sCD146 and VEGF on CD146-negative glioblastoma cells. Supplementary Figure 8: sCD146 binds integrin αvβ3 on U373 cells. Supplementary Figure 9: Soluble CD146 induces EMT in U118 cells transfected with integrin αvβ3. Supplementary Figure 10: Knocking-down integrin αvβ3 inhibits sCD146-induced EMT in U87 cells. Supplementary Figure 11: sCD146 mediated its effects on U87 cells through a signalosome containing CD146, αvβ3, and VEGFR2. Supplementary Figure 12: sCD146 mediates its effects on U373 cells through a signalosome containing CD146, αvβ3, and VEGFR2. Supple [file 40478_2022_1451_MOESM1_ESM.docx]

**Soluble CD146, a biomarker and a target for preventing resistance to anti-angiogenic therapy in glioblastoma**

**Joshkon et al**

**SUPPLEMENTARY METHODS**

**Peptides and antibodies**

Recombinant human sCD146 (rsCD146) and VEGF-165 (rVEGF) were obtained from Biocytex (Marseille, France) and Invitrogen (PHC9391), respectively. Recombinant human integrin αvβ3 was from R&D (cat# 3050-AV-050) and recombinant RGD peptide was from Santa Cruz (sc-201176). Antibodies used in this study are listed in supplementary table 2.

**Plasmids, siRNA and cell transfection**

The pEF1-alpha-V (#27290) and pcDNA3.1-beta-3 (#27289) plasmids encoding integrin subunits αv and β3, respectively, were purchased from Addgene. The corresponding empty vectors were used as a negative control. The small interfering RNA (siRNA) targeting integrin αL (AM16708), integrin β2 (HSS105562), integrin αv (HSS105554) and integrin β3 (HSS105565) gene transcripts and the silencing negative control (AM4611) were from Invitrogen. Cell transfection was carried out using Polyplus® jetPrime reagent (Ref. 114-15) according to the manufacturer’s instructions. For integrin αvβ3 co-transfection experiments, equal amount of pEF1 and pcDNA3.1 vector was added.

**Western-blot and Immunoprecipitation assays**

Briefly, cells were grown on plates and treated as a function of the experiment, then washed in PBS, scraped off the plates and extracted with 300 μl of ice-cold RIPA buffer (150 mM NaCl, 50 mM Tris HCl, pH 7.4, 2.4 mM EDTA, 1% Nonidet P40, 0.5 mM phenylmethylsulfonyl fluoride, supplemented with protease and phosphatase inhibitor (A32959)) for 30 min at 4°C. After centrifugation (12,000 g, 10 min, 4°C) to eliminate cell debris and nuclei, the Pierce^TM^ BCA protein assay kit was used to quantify protein concentration. 25 µg of proteins were subjected to SDS-PAGE (NuPAGE™ 4 to 12%, Invitrogen), and then transferred to a nitrocellulose membrane using iBlot2 system, Invitrogen. The membranes were blocked in TBST-5% BSA for 1h at room temperature and then probed separately with primary antibodies overnight at 4°C. The blots were finally incubated with HRP-conjugated secondary antibody for 1 hour at room temperature and protein bands were revealed using ECL substrates (Invitrogen). Mild stripping buffer was used to strip membranes and re-probe with another primary antibody. Immunoblotting on mice brains was done on 100 mg of tissues. Briefly, mice brains were snap frozen in liquid nitrogen, minced, and three fractions of 100 mg each from the same brain was mixed with RIPA for 30 minutes on ice.

For immunoprecipitation, non-denaturing lysis buffer (20 mM Tris HCl pH 8, 137 mM NaCl, 1% Nonidet P-40 (NP-40), 2mM MnCL_2,_ 1mM CaCL_2_) was used in all experiments unless otherwise specified. Briefly, 5 µg of primary antibody was added to cleared whole cell lysate and kept overnight on a rotator in the cold room. The next day, Dynabeads™ Protein G was added for 1 hour at 4°C. The beads were then trapped with a magnetic field, washed trice with PBS^+/+^ and finally resuspended in RIPA buffer. The complex was heated at 95°C for 10 minutes and loaded on SDS-PAGE. In immunodepleting experiments, the same procedure was followed except the depleted fraction was preserved at -80°C until use.

**ELISA experiments**

ELISA kits used to specifically detect human sCD146 and human VEGF were from BioCytex (cat#7501) and BioLegend (cat#446507), respectively. In-house ELISA was developed to detect the interaction between sCD146 and integrin αvβ3. Briefly, 0.5 µg of either recombinant sCD146 or recombinant integrin αvβ3 was added into the wells of a 96-well plate. After overnight incubation at 4°C, wells were thoroughly washed with PBS-Tween 0.05%, blocked with 4% BSA, and then the reciprocal interacting recombinant proteins were added for 1 hours at room temperature. After several washes, wells were probed with anti-integrin αvβ3 or anti-sCD146. The plate was then washed and a secondary antibody coupled to HRP was added for 30 minutes at room temperature. The interaction was revealed by adding peroxide substrate, TMB, and incubating for 5 minutes at room temperature. The reaction was finally stopped with sulfuric acid and absorbance was read at 450nm.

**Immunofluorescence experiments**

Cells were seeded in Lab-Tek™ II Chamber Slide (Invitrogen) at 80% confluency and were then transfected with plasmids encoding or not integrin αv and β3 subunits. For siRNA experiments, cells were seeded at 50% confluency and transfected after 24 hours with integrin β3 siRNA. 48 hours post-transfection, cells were washed twice with PBS, serum-starved for 2 hours, and recombinant sCD146-FITC protein was then added for 30 minutes at 37°C. An irrelevant IgG-FITC was used as a negative control. The cells were then washed twice with PBS, fixed with 4% PFA, and nuclei were counterstained with DAPI (BioLegend). The slides were finally mounted in ProLong Glass antifade (Invitrogen). Images were visualized using Leica inverted microscope (Leica DFC9000 GTC).

**Flow cytometry experiments**

Flow cytometry experiments were conducted with a Gallios Flow cytometer (Beckman Coulter, CA, USA). Briefly, all detached cells were labeled with the antibody or isotype matched control antibody directly coupled to fluorescent dye (10 μg/ml) (Supplementary Table 2) for 30 min at 4°C. After washing, samples were analyzed by flow cytometry. Dead cells were omitted from analysis using LIVE/DEAD™ Fixable Aqua dye (Invitrogen). Isotype controls were used to exclude false positive cells. Results were analyzed with Kaluza software (Beckman Coulter, CA, USA).

**Reverse Transcription-quantitative PCR (RT-qPCR)**

mRNA expression of several genes was studied by reverse transcription (RT) and quantitative polymerase chain reaction (qPCR). Cells were incubated in experimental conditions. Total RNA was extracted using the ReliaPrep™ RNA Cell Miniprep System (Promega), according to the manufacturer's instructions. DNA was digested with DNase (RNase-Free DNase Set, Qiagen) to secure complete DNA removal. Reverse transcription using random primers was performed on 1 µg of total RNA of each sample using Superscript II reverse transcriptase (Invitrogen). 50 ng of cDNA were then subjected to RT-qPCR using pre-designed TaqMan primers (Invitrogen). The housekeeping gene GAPDH was used as an internal control to normalize gene expression. The relative expression level of a particular gene was evaluated using the 2−ΔΔCt method. Each point was run in triplicate.

**Cell migration assays**

Cells were seeded in 24-well plates at 90% confluency. The following day, once at confluency, cells were serum-starved for 4 hours and then a hole was made in the center of each well. A reproducible hole was performed using a 200 μl pipet tip. After 3 washes with minimal medium, cells were stimulated with either rsCD146, VEGF, or conditioned media depleted or not from these factors. The first image acquired was assumed to be at time T0. Images were then automatically taken every 2 hours for up to 8 hours in environmentally controlled conditions (37°C, 5% CO_2_) on Leica microscope. The extent of cell migration was calculated according to the following formula: 100-((A1/A0) *100) whereby A0 is the area of the hole at time T0 and A1 is that after 8 hours of migration as calculated using ImageJ software.

**Cell Proliferation assays**

5×10^3^ U87, U118 or U373 cells were seeded in 96-well plates for 24 hours. Cells were serum-starved for 4 hours and then exposed to different treatments as described in the figure legend. Cell proliferation was assayed by WST-1 proliferation kit (Sigma-Aldrich) after 48 hours of stimulation. WST-1 cell proliferation assay**utilizes a tetrazolim salt WST-1[2-(4-Iodophenyl)-3-(4-nitrophenyl)- 5-(2,4-disulfophenyl)-2H-tetrazolium].** WST-1 produces a highly water-soluble formazan upon metabolically active cells, allowing a direct and user-friendly colorimetric measurement of cell proliferation. Briefly, 10µL of WST-1 reagent was added into the medium and incubated for 2 hours at 37°C before measuring the absorbance value at 450nm using the microplate reader GloMax (Promega).

**Transwell invasion assays**

Invasion assay were conducted to examine the invasive capacity of the cells. Briefly, cells were stimulated for 48 hours with different treatments as described in the figure legend and then 25,000 were seeded in upper transwell chambers (8 µm pore size; Corning) previously coated with serum-reduced Matrigel (Corning). Complete media containing 10% FCS was added to the lower chamber. After 48 hours, non-invaded cells were scrapped off while cells on the other face of the insert were fixed with 4% paraformaldehyde and stained with 0.5% crystal violet. The number of cells from three separate fields in each well was counted. Each assay was performed in triplicate and representing images are shown.

**Crispr/Cas9 deletion of genes**

The vector pSpCas9(BB)-2A-Puro (PX459) V2.0, expressing both Cas9 and sgRNA scaffold was purchased from Addgene. gRNAs targeting the genes CD146-Exon 6, ITGB3-Exon 1, and VEGFR2-Exon 1 were designed using CRISPOR tool [(http://crispor.org/)](http://crispor.org/) and selected based on minimal off-target score. The 5’-3’ oligo sequences are as follow; CD146-Exon 6 sense: CACCGTCAACTACCGGCTGCCCAGT, CD146-Exon 6 anti-sense: AAACACTGGGCAGCCGGTAGTTGAC, ITGB3 Exon 1 sense: CACCGAGGCGGACGAGATGCGAGCG, ITGB3 Exon 1 anti-sense: AAACCGCTCGCATCTCGTCCGCCTC, VEGFR2 Exon 1 sense: CACCGTGCTGCTGGCCGTCGCCCTG, VEGFR2 Exon 1 anti-sense: AAACCAGGGCGACGGCCAGCAGCAC. The PX459 plasmid was digested with BbsI and gel purified using the Wizard SV Gel and PCR Clean-Up System (Promega). A pair of oligos for each target site was phosphorylated, annealed and ligated into linearized pX459 vector for generating gRNA-expressing plasmid. The resulting plasmids were separately transformed into JM109 competent cells and minipreps were carried out on the resulting colonies. U87 and U373 cells were then transfected with 10 µg of gRNA-containing plasmids using jetPrime (PolyPlus) according to the manufacturer's protocol. Cells were cultured for 10–14 days with puromycin (2 µg/mL) and single cell-derived colonies were finally harvested and amplified.

Genomic DNA from the CRISPR-Cas9 modified cells was isolated and sequences encompassing sgRNA-targeted regions were PCR-amplified (Platinum Taq DNA Polymerase, Invitrogen) using primers flanking the targeted sequence of the sgRNAs. Amplified fragments were then sequenced (Illumina HiSeq 2500).

**Experiments on animals and imaging**

Four to five weeks old NMRI-Foxn1nu female mice (Janvier-labs) were used. For anesthesia, either isoflurane gas or ketamine/xylazine IP injection was used.

In U87 ectopic tumor model, xenograft of the human glioblastoma cell line U87 was produced by subcutaneously injecting 10^6^ cells into the back of NMRI-foxn1 nude mice. When tumors reached 20 mm^3^, peri-tumoral administration of irrelevant human IgG1, humanized anti-sCD146 “mucizumab”, anti-VEGF “bevacizumab”, or a combination of both humanized antibodies began at a dose of 10 µg, twice a week for 4 weeks. Tumor size was measured once a week with a caliper and tumor volume was determined according to the equation: ((length*width*height) *π/6).

In U87 orthotopic tumor model, orthotopic injections of U87 cells (10^5^ in 3µL PBS) were performed using a stereotactic frame (Stoelting) at 2 mm on the right of the medial suture, 1 mm in front of the bregma, and at a depth of 2.5 mm. Cells were injected on the same day into 4 weeks old NMRI-Foxn1nu female mice. Ten days post-implantation, mice were randomized and regrouped into i) control IgG, ii) bevacizumab, iii) mucizumab, and iv) mucizumab + bevacizumab receiving groups. Antibodies (5mg/kg) were retro-orbitally administered and thereafter every two days for up to two weeks. Animals were then anesthetized, perfused with PBS followed by 4% PFA, and brains were finally isolated. At least four mice were used in each group. The brains were then fixed in PFA for 24 hours, saturated in increasing concentrations of sucrose, and stored in isopentane at -80°C until use.

In representative animals of the U87 orthotopic tumor model, MicroPET/CT acquisitions were performed on a NanoscanPET/CT camera (Mediso, Budapest, Hungary) using 86Ga-RGD. Radioactivity was injected intravenously in the retro-orbital sinus. Mice were maintained under 2% isoflurane anesthesia during acquisition. Static microPET imaging was performed 1 h after each radiotracer injection, during 20 min.

**Immunohistochemistry**

At the end of the experiments, animals were perfused at room temperature with PBS then with paraformaldehyde (PFA) 4% at room temperature before sacrifice. Brains were then removed and incubated for 24h at 4°C in PFA 4%, then for 8h at 4°C in sucrose 20%, and finally for 3 days at 4°C in sucrose 30%. Brains were then stored at -80°C in isopentane. Cryopreserved brain tissues were serially cut into 10 μm-thick coronal sections using a Cryostat (Leica, CM 3050S, Wetzlar, Germany) at -20°C. For immunohistochemistry, brain sections were brought out of -80°C and allowed to reach room temperature. Slides were then rehydrated in a series of TBS bath and blocked in TBS-2% BSA for 1 hour at room temperature. The brain sections were then incubated with HRP-conjugated primary antibody directed against human CD146 (clone COM7A4-HRP, 1:20) overnight at 4°C. Staining was visualized by using 3, 30-diaminobenzidine (DAB), followed by hematoxylin counterstaining (Sigma-Aldrich). Slides were scanned using Leica microscope (Leica DFC9000 GTC). Images panels were prepared using Fiji or ImageJ software. To estimate tumor volume, Immunohistochemistry Image Analysis Toolbox [Shu J, Qiu G, Ilyas M (2014) Immunohistochemistry (IHC) Image Analysis Toolbox. 28] was used in ImageJ software. The tumor area in each section was calculated, and then the volume was estimated by multiplying with tumor thickness.

**Patient cohort**

We retrospectively included all adult patients referred to our institution for recurrent *IDH* wild-type glioblastoma who received bevacizumab at the dose of 15 mg/kg every 3 weeks in association with carmustine at the dose of 150 mg/m² every 6 weeks for whom plasma samples were available before bevacizumab administration (D1) and after 21 days from the first bevacizumab administration (D21). Clinical and imaging evaluations were performed every 3 weeks and 6 weeks respectively. Treatment responses and disease progression were reviewed using the RANO criteria [Wen PY, Macdonald DR, Reardon DA et al (2010) Updated response assessment criteria for high-grade gliomas: response assessment in neuro-oncology working group. J Clin Oncol 28:1963–1972]. All patients provided written informed consent in accordance with institutional, national guidelines and the Declaration of Helsinki.

Plasma samples were collected at baseline, before bevacizumab administration (D1), and after the first bevacizumab administration (D21).

**Statistical analysis**

The data are expressed as mean values ± SEM.

Categorical variables were presented as frequencies and percentages, continuous variables as median and range. Overall survival (OS) was defined to be the time from first bevacizumab administration to death from any cause, censored at the date of last contact. Progression-Free Survival (PFS) was the time from first bevacizumab administration to documented progression or death, censored at the date of the last documented disease evaluation. Kaplan-Meier method was used to estimate survival curves. Log-rank tests were used for univariate comparisons. sCD146 plasmatic levels were dichotomized by their median values for survival analyses. Mann-Whitney U-test or Kruskal Wallis test were used to compare quantitative and qualitative values; qualitative values were analyzed by Fisher exact test and Chi 2 test.

Statistical analysis was performed using Prism software (GraphPad Software Inc., San Diego, CA, USA) or SPSS ® v22. The variance between the different groups to be compared was estimated before statistical analysis. Significant differences between two groups were determined using the unpaired, 2-tailed, Mann-Whitney U test. A value of P ≤ 0.05 was considered to be significant.

**SUPPLEMENTARY FIGURES AND TABLES**

|  | Progression-Free Survival | Overall Survival |
| --- | --- | --- |
| Age (cutoff: median) | 0.320 | 0.124 |
| KPS (< 80 versus ≥ 80) | 0.122 | 0.818 |
| Steroid dose (cutoff: median) | 0.092 | 0.353 |

**Table 1: Absence of significant impact of age, KPS and steroid dose on progression-free survival or overall survival.**

**The effects of age, Karnofsky score,** a system allowing medical doctors to evaluate a patient's ability to survive to chemotherapy **(KPS),** and steroid dose received by the patient, were tested on progression-free survival (PFS) and overall survival (OS) by univariate analyses. Statistical significances are given.

| Antibody | Manufacturer | Reference | Dilution | Application |
| --- | --- | --- | --- | --- |
| Phospho-VEGFR2 (Tyr1054), Clone D1W | Merck | 04-894 | 1:1000 | WB |
| Anti- VEGF Receptor 2 antibody, Clone A3 | Santa Cruz | sc-6251 | 1:1000 | WB |
| Anti-VEGF Receptor 2 antibody | Abcam | ab39638 | 1:1000 | WB |
| Phospho-FAK (Tyr397) | ThermoFisher | 44-624G | 1:1000 | WB |
| FAK Polyclonal Antibody | ThermoFisher | AHO0502 | 1:1000 | WB |
| Phospho-Integrin β3 Antibody (pY759.7A) | Santa Cruz | sc-136458 | 1:1000 | WB |
| Integrin β3 Antibody (D-11) | Santa Cruz | sc-365679 | 1:1000 | WB |
| Anti-human CD51 Antibody | Biolegend | 327902 | 1:750 | WB |
| CD51/CD61 Monoclonal Antibody (23C6) | eBioscience | 14-0519-82 | 5µg | IP/ELISA |
| Anti-CD146 COM7A4 (human specific) | BioCytex | 5040-P | 1:750 | WB/ELISA |
| Anti-CD146, S-Endo1 | BioCytex | 5050-P | 5µg | IP |
| Anti-CD146 antibody [EPR3208] | Abcam | ab75769 | 1:1000 | WB |
| Phospho-Tyr Antibody (PY99) | Santa Cruz | sc-7020 | 1:750 | WB |
| Phospho-Akt (Ser473) Antibody | CST | #9271 | 1:1000 | WB |
| Anti-Akt Antibody | CST | #9272 | 1:1000 | WB |
| Phospho-p38MAPK (Thr180/Tyr182) Antibody | CST | #9211 | 1:1000 | WB |
| Anti-p38 MAPK Antibody | CST | #9212 | 1:1000 | WB |
| Phospho-ERK1/2 (Thr202/Tyr204) Antibody | BioLegend | 675502 | 1:1000 | WB |
| Anti-ERK 1/2 Antibody (C-9) | Santa Cruz | sc-514302 | 1:1000 | WB |
| N-Cadherin (D4R1H) XP® Rabbit mAb | CST | #13116 | 1:1000 | WB |
| E-Cadherin (24E10) Rabbit mAb | CST | #3195 | 1:1000 | WB |
| Vimentin (D21H3) XP® Rabbit mAb | CST | #5741 | 1:2000 | WB |
| Snail (C15D3) Rabbit mAb | CST | #3879 | 1:1000 | WB |
| Slug (C19G7) Rabbit mAb | CST | #9585 | 1:1000 | WB |
| Nanog Antibody | R&D | AF1997 | 1:1000 | WB |
| Oct-3/4 Antibody (C-10) | Santa Cruz | sc-5279 | 1:1000 | WB |
| Sox-2 Antibody (A-5) | Santa Cruz | sc-365964 | 1:1000 | WB |
| β-Actin Antibody | CST | #4967 | 1:2000 | WB |
| Anti-EpCAM Antibody (C-10) | Santa Cruz | sc-25308 | 1:800 | WB |
| Anti-MAX antibody [73C5a] | Abcam | ab53570 | 1:1000 | WB |
| Anti-β-Amyloid, 1-16 Antibody | BioLegend | 803004 | 1:1000 | WB |
| Goat anti-Mouse IgG (H+L) Secondary Antibody, HRP | ThermoFisher | 31430 | 1:2500 | WB/ELISA |
| Goat anti-Rabbit IgG (H+L) Secondary Antibody, HRP | ThermoFisher | 31460 | 1:2500 | WB |
| Rabbit anti-Goat IgG (H+L) Secondary Antibody, HRP | ThermoFisher | 81-1620 | 1:2500 | WB |
| FITC anti-human CD51/61 Antibody | BioLegend | 304404 |  | FC |
| FITC Mouse IgG1, κ Isotype Ctrl Antibody | BioLegend | 400108 |  | FC |
| APC anti-human CD146 Antibody | BioLegend | 361016 |  | FC |
| APC Mouse IgG1, κ Isotype Ctrl (FC) Antibody | BioLegend | 400122 |  | FC |
| PE anti-human CD309 (VEGFR2) Antibody | BioLegend | 359904 |  | FC |
| PE Mouse IgG1, κ Isotype Ctrl Antibody | BioLegend | 400112 |  | FC |
| Angiomotin Antibody (B-4) FITC | Santa Cruz | sc-166924 |  | FC |
| FITC Mouse IgG2b, κ Isotype Ctrl Antibody | BioLegend | 400310 |  | FC |
| Human IgG1, whole molecule | Jackson ImmunoResearch Laboratories | 009-000-003 | 5 mg/kg | In vivo |
| Bevacizumab | Roche |  | 5 mg/kg | In vivo |
| Mucizumab |  |  | 5 mg/kg | In vivo |

**Table 2: references and application condition of the different antibodies used in the study**

**
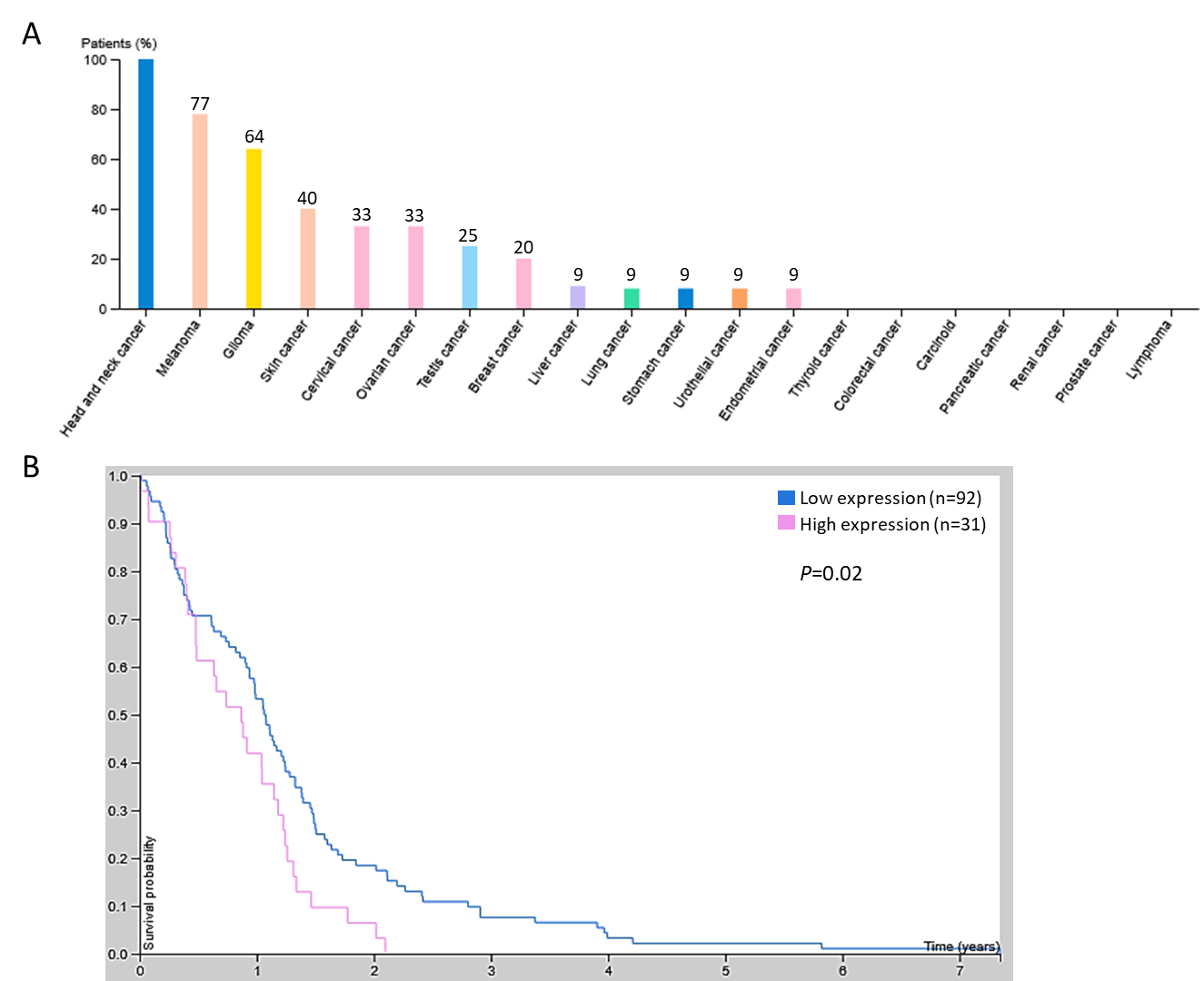
**

**Figure S1: Expression of CD146 on various types of cancers and effect on overall survival in patients with GBM. (A**) Percentage of patients with high or medium CD146 expression level as revealed by immunohistochemistry analysis. **(B)** Kaplan-Meier plot showing overall survival of 123 patients who died of GBM as function of low or high CD146 expression. The data were obtained from the human protein Atlas database.

**
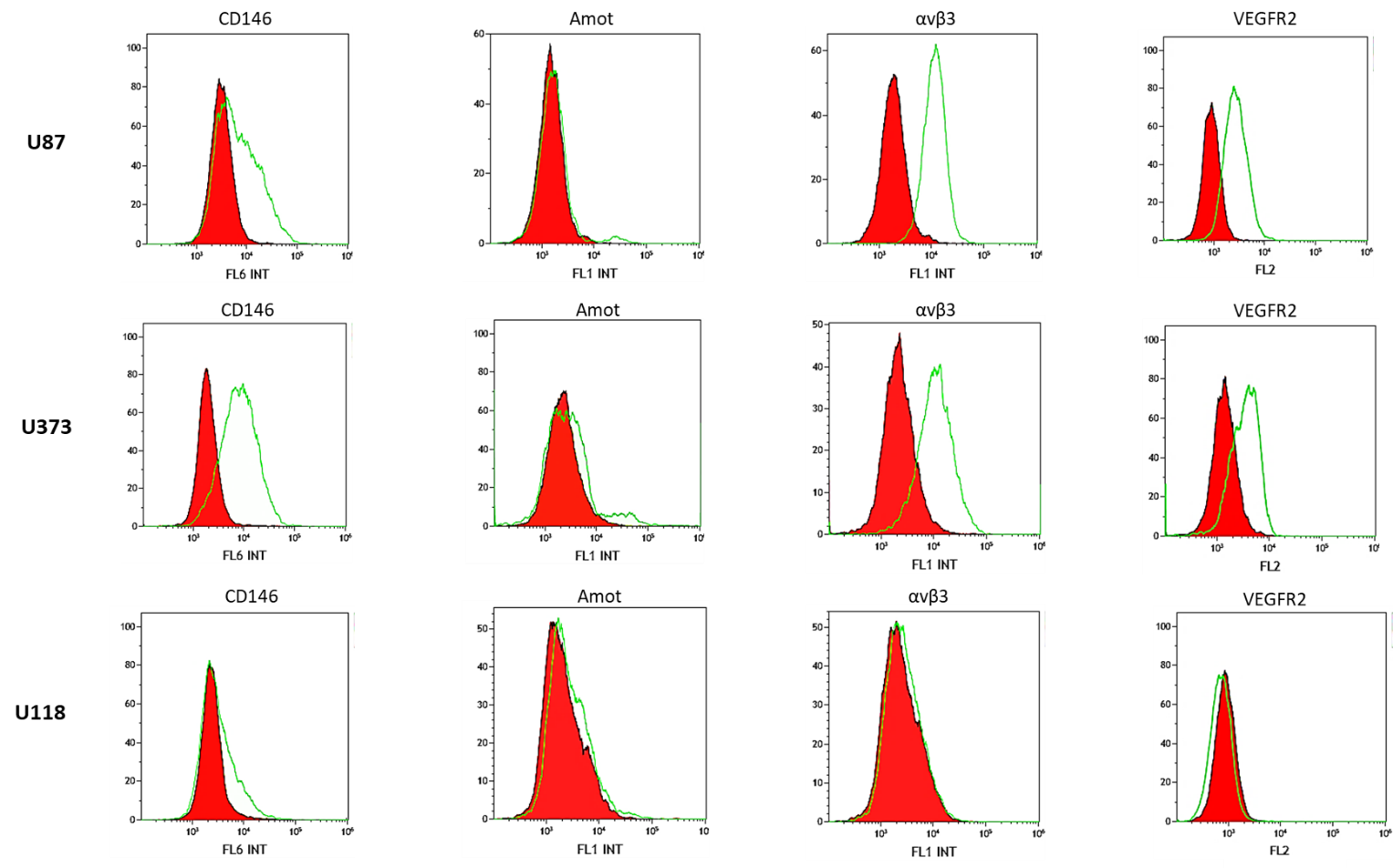
**

**Figure S2: Glioblastoma cell lines characterization.** Surface expression of CD146, VEGFR2, integrin αvβ3, and Angiomotin (Amot) on glioblastoma cell lines U87, U373, and U118 were assessed by flow cytometry. Red histogram corresponds to isotype control antibody and green histogram to antibody of interest.

**Figure S3: Concentration of VEGF and sCD146 in the culture media of U87, U373, and U118 glioblastoma cell lines.** Elisa was performed using cultured media from the three glioblastoma cell lines. The concentrations are normalized to total cell number.

Average of 3 experiments is shown, each point was run in triplicate.

**
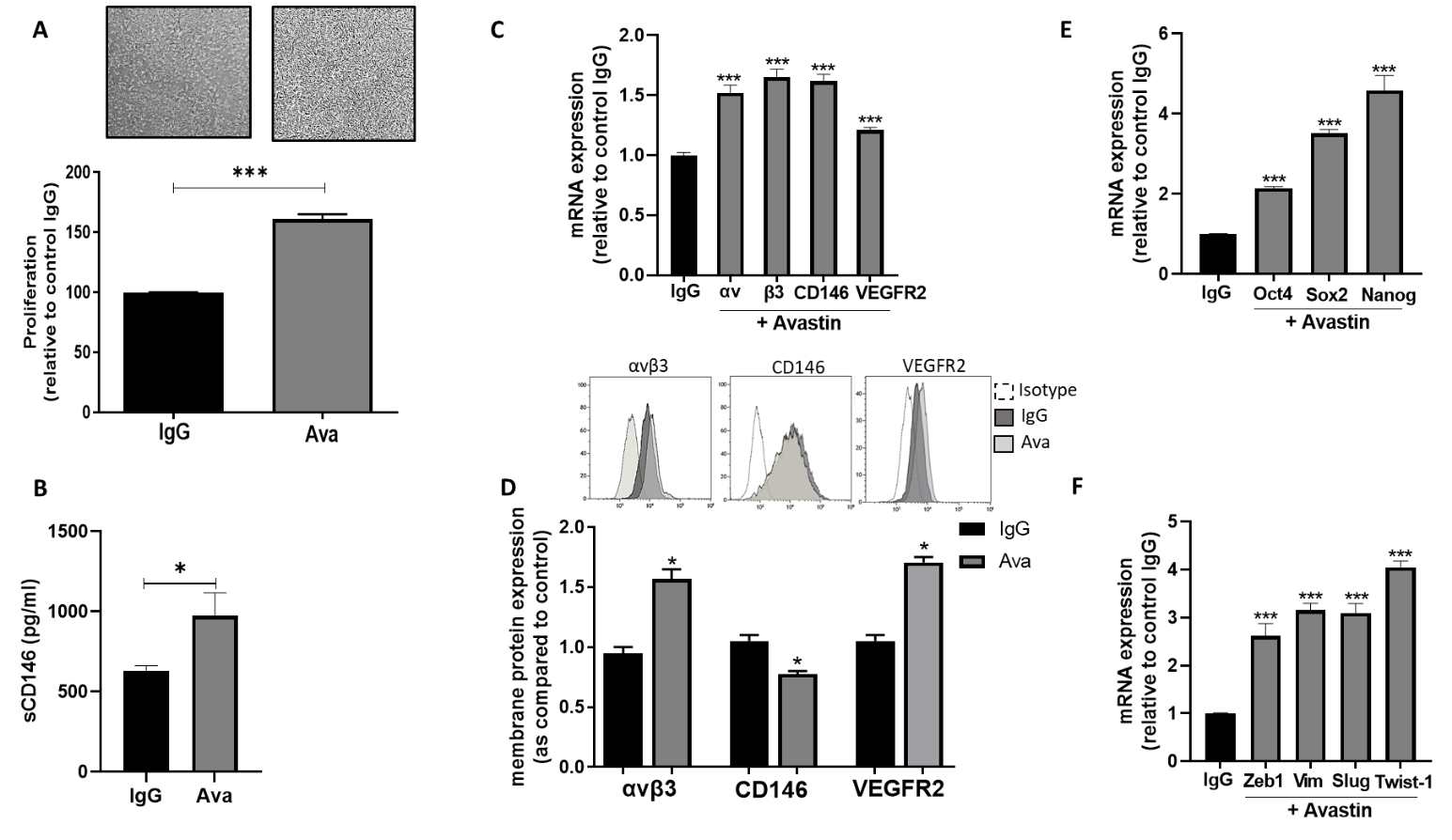
**

**Figure S4: Avastin induces proliferation, sCD146 secretion, and EMT/CSC markers in U373 cells.** In-vitro challenging of U373 cells with Avastin (100 µg/mL) enhances cell proliferation **(A)** and increases sCD146 secretion **(B)** as compared to IgG treated cells. Avastin-challenged U373 cells upregulate CD146, VEGFR2 and integrin subunits αv and β3 gene transcription **(C)** and their membrane expression, except for CD146 **(D)** as revealed by q-PCR and flow cytometry, respectively. U373 challenging with Avastin potently induces expression of markers related to CSC **(E)** and EMT **(F)** at the mRNA level as compared to control IgG treated cells.

Average of 5 experiments is shown; **P<0.01, ***p<0.001, experimental vs control.

**
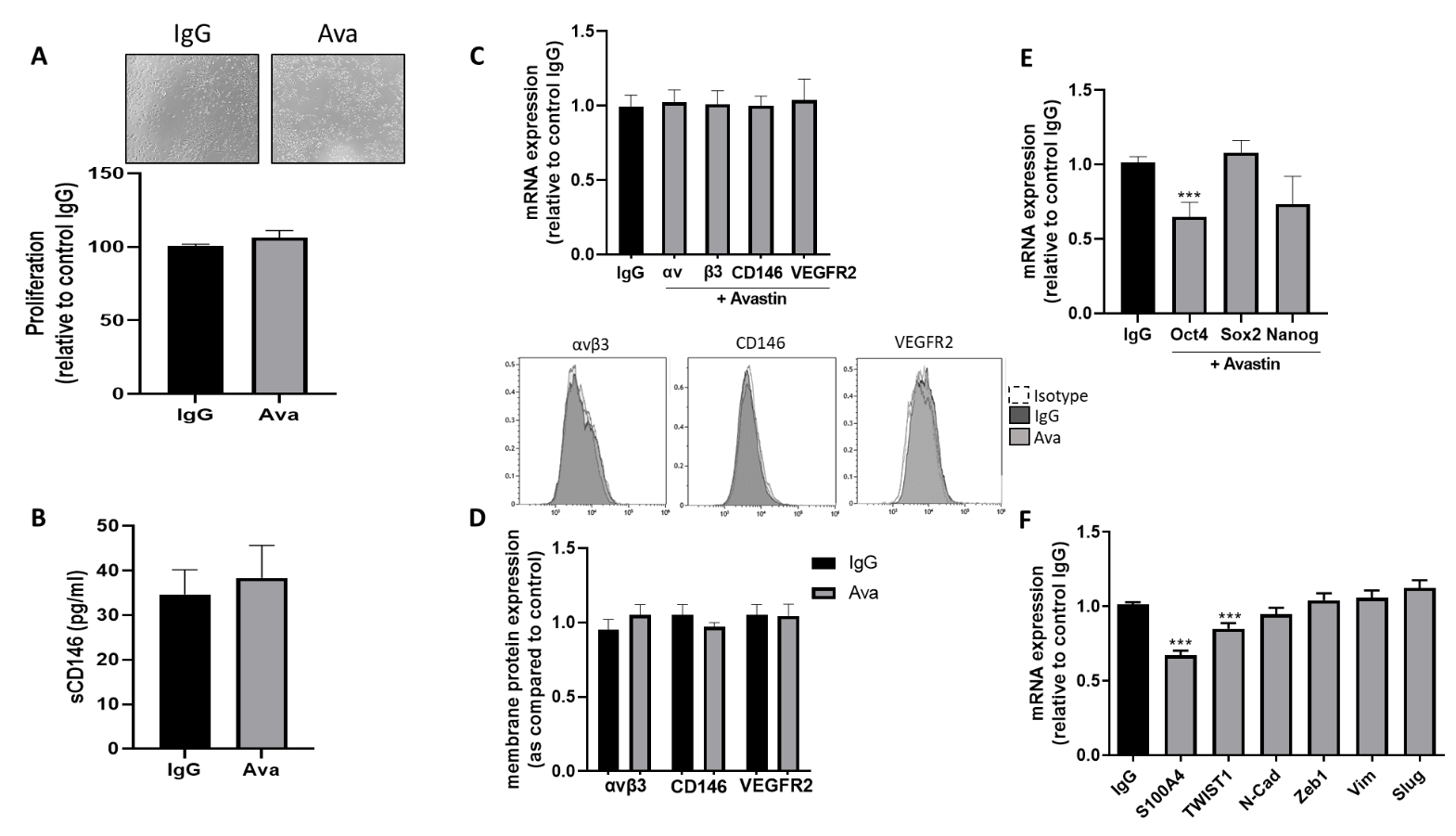
**

**Figure S5: Avastin has no effect on CD146-negative U118 glioblastoma cells.** The effect of in-vitro challenging of U118 cells with Avastin (100 µg/mL) on cell proliferation **(A)** and sCD146 secretion by the cells **(B)** as compared to IgG treated cells are shown. Gene expression of CD146, VEGFR2 and integrin subunits αv and β3 in the Avastin-challenged U118 cells were assessed by q-PCR **(C)** and their membrane expression by flow cytometry **(D).** The expression of markers related to CSC **(E)** and EMT **(F)** at the mRNA level in Avastin-challenged U118 cells is shown.

Average of 3 experiments; ***p<0.001, experimental vs control.

**
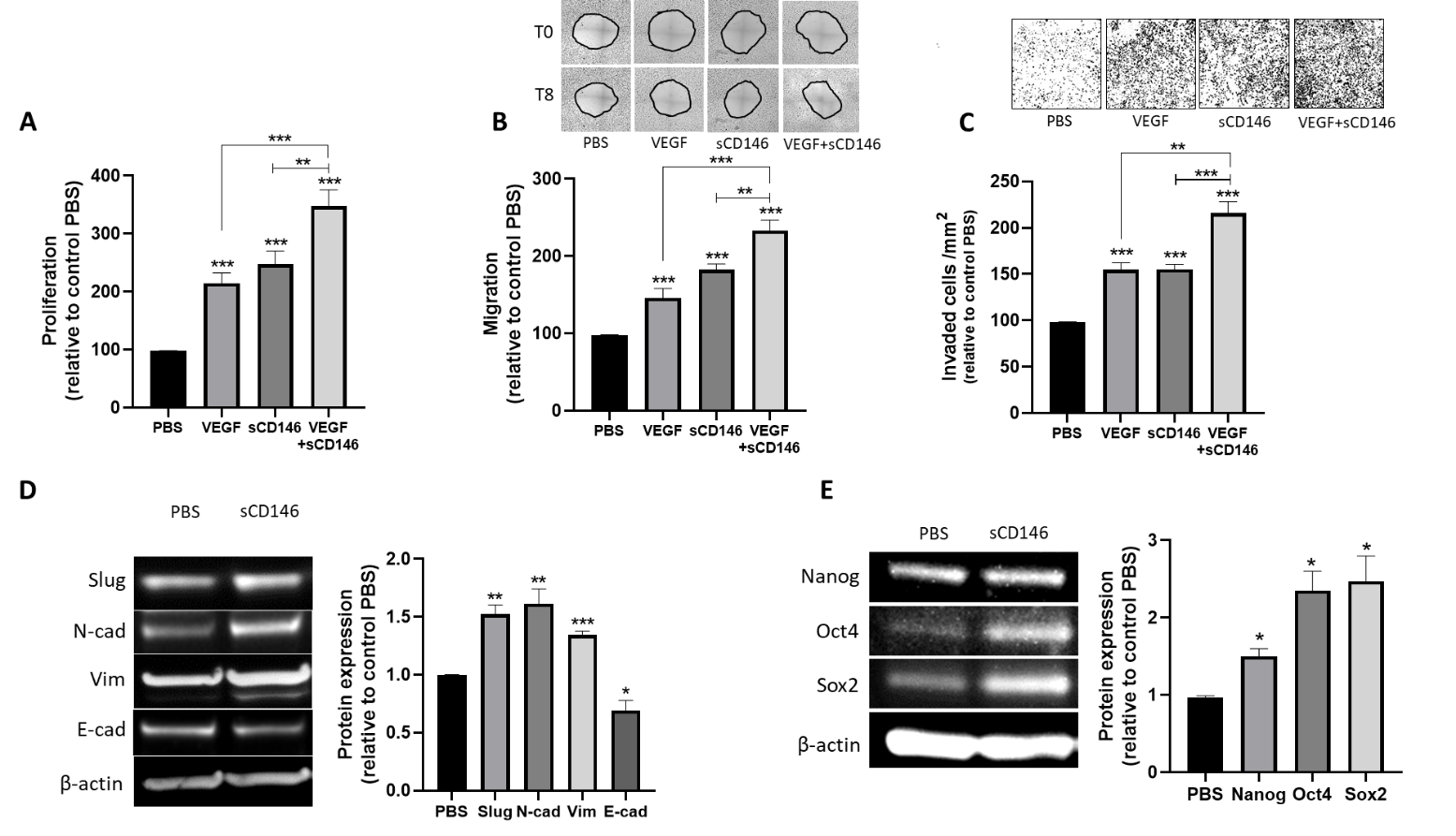
**

**Figure S6: sCD146 induces U373 cell proliferation, migration and invasion in-vitro and promotes CSC and EMT markers.** U373 cells were treated with 100 ng/ml of VEGF, rsCD146 or combination of both molecules for 48 h and cell proliferation **(A)**, migration **(B)** and invasion **(C)** were determined. EMT **(D)** and CSC **(E)** markers were also examined after 48h of treatment with 100 ng/ml sCD146. Representative blots from 5 experiments are shown.

Average of 3 experiments; *P<0.05, **P<0.01, ***p<0.001, experimental vs control.

**
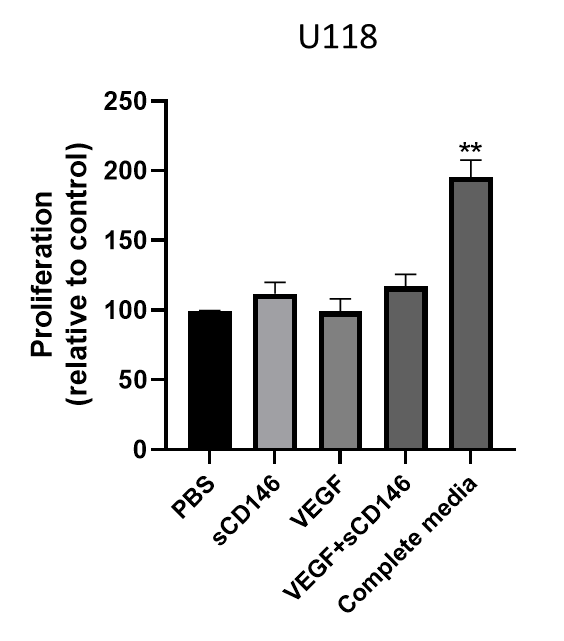
**

**Figure S7: Effect of sCD146 and VEGF on CD146-negative glioblastoma cells.** U118 cells were treated with sCD146 and/ or VEFG and the cell proliferation was measured.

Average of 3 experiments is shown; ** P<0.01, experimental vs control.

**
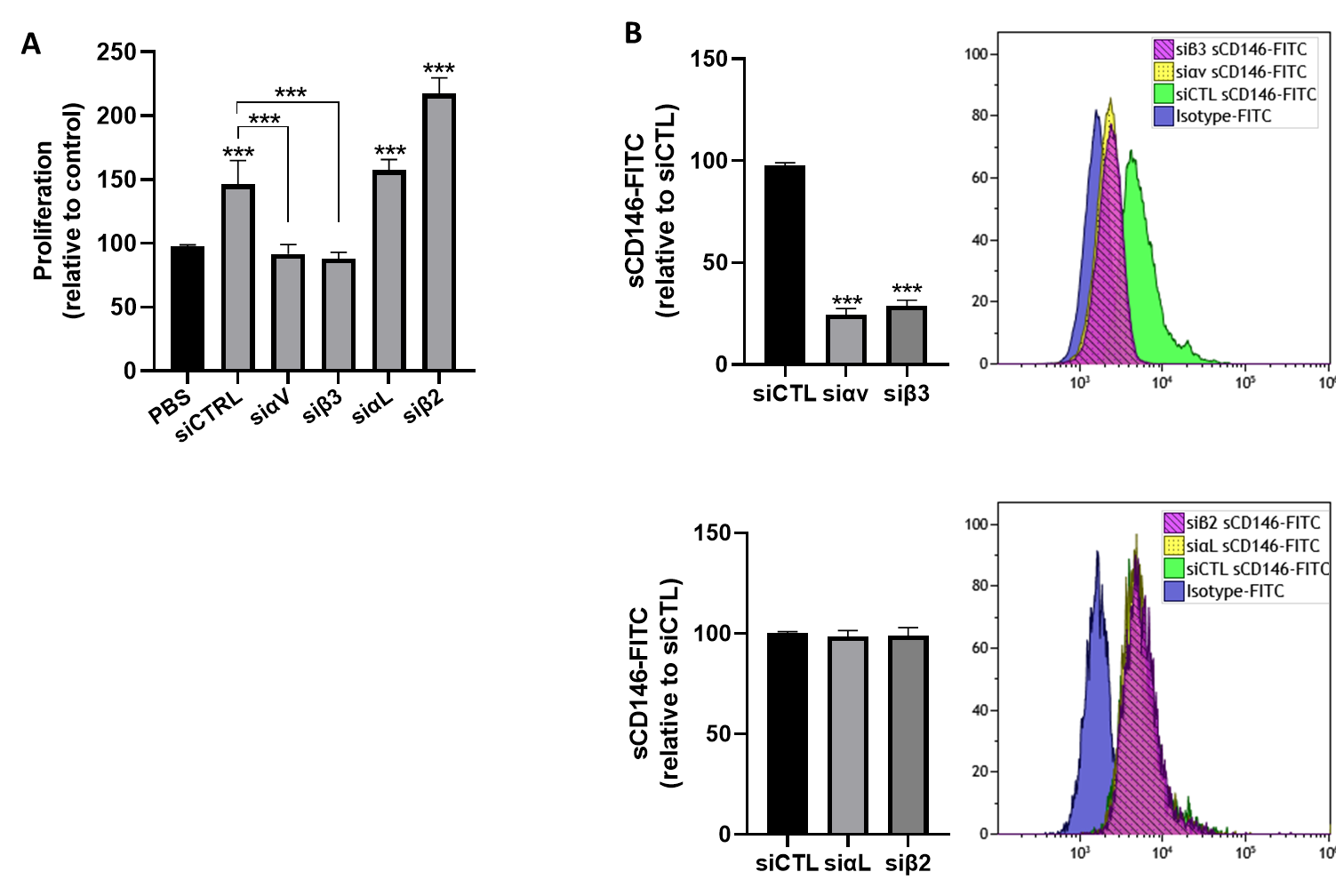
**

**Figure S8: sCD146 binds integrin αvβ3 on U373 cells.**

The effect of silencing RNA targeting αv, β3, αL and β2 was analyzed on sCD146 proliferative effect on U373 cells **(A)**. U373 cells were transfected with siRNA targeting αv, β3, αL and β2 and sCD146-FITC binding was determined by flow cytometry **(B)**.

Average of 3 experiments is shown; ***p<0.001, experimental vs control.

**
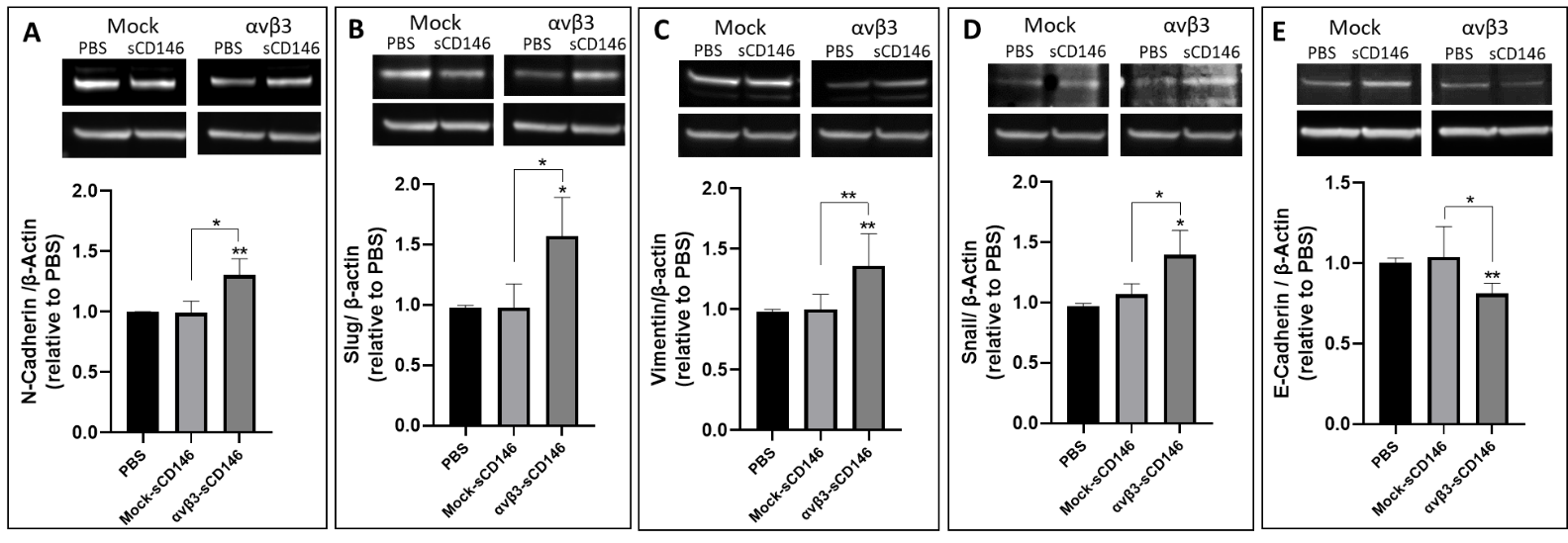
**

**Figure S9: Soluble CD146 induces EMT in U118 cells transfected with integrin αvβ3.**

U118 cells were transiently co-transfected with plasmids encoding αv and β3 integrin subunits and then treated with sCD146. Immunoblots using anti-N cadherin**(A),** anti-slug **(B),** anti-vimentin **(C),** anti-snail **(D),** anti-E cadherin **(E)** antibodies are shown.

Average of 5 experiments; *P<0.05, **P<0.01, experimental vs control.

**
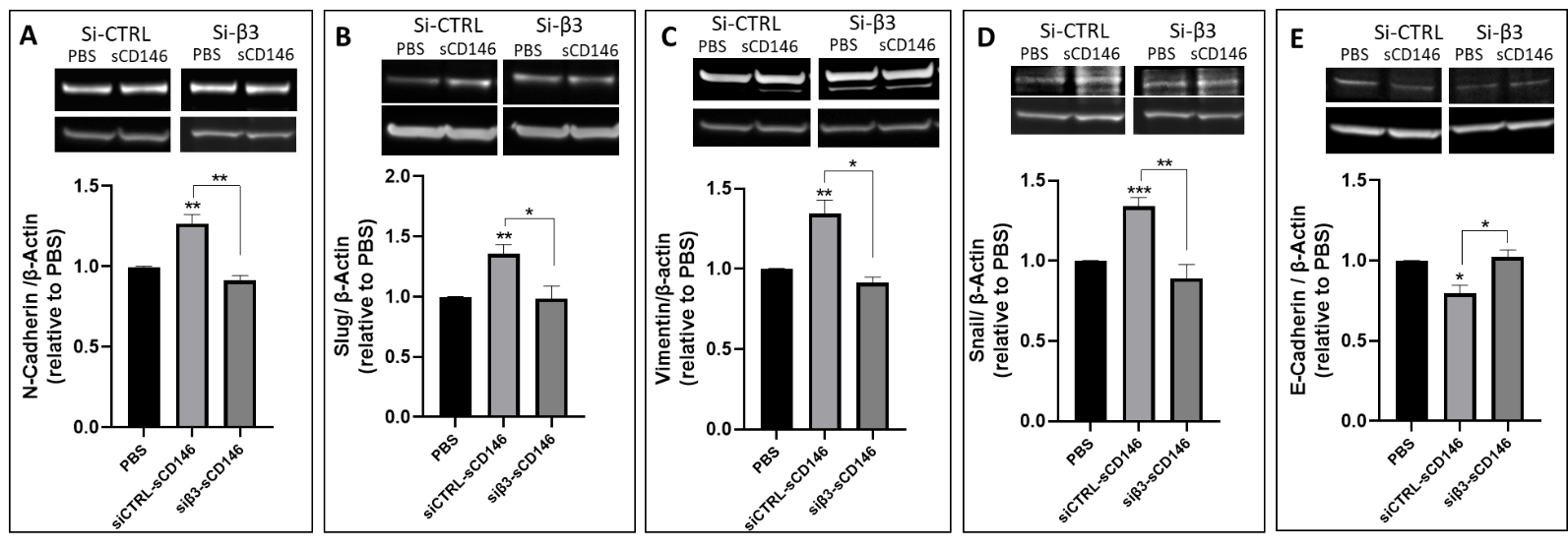
**

**Figure S10: Knocking-down integrin αvβ3 inhibits sCD146-induced EMT in U87 cells.**

U87 cells were transfected with siRNA targeting integrin β3 subunit and then treated with sCD146. Immunoblots using anti-N cadherin**(A),** anti-slug **(B),** anti-vimentin **(C),** anti-snail **(D),** anti-E cadherin **(E)** antibodies are shown.

Average of 5 experiments; *P<0.05, **P<0.01, experimental vs control.


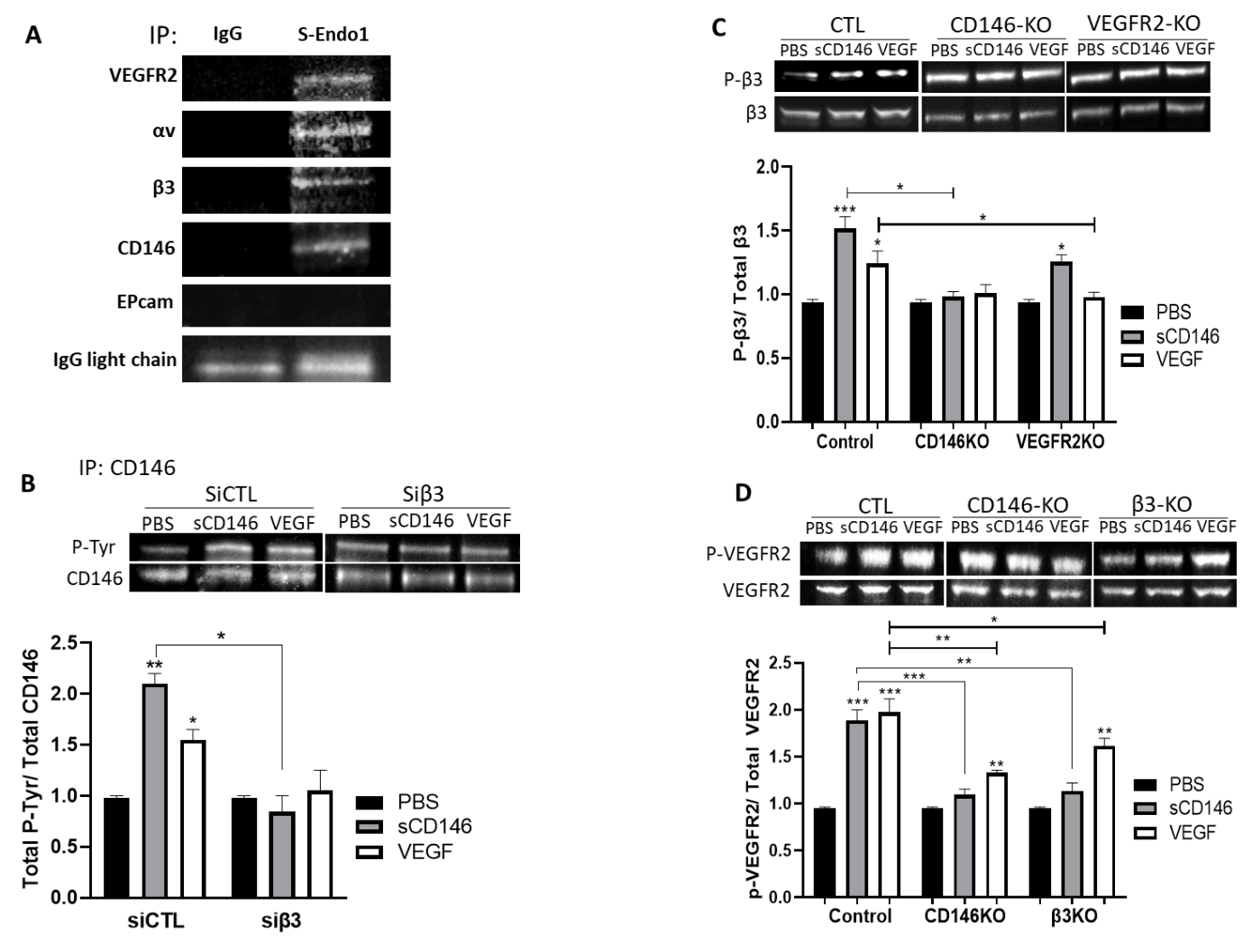


**Figure S11: sCD146 mediated its effects on U87 cells through a signalosome containing CD146, αvβ3, and VEGFR2.**

Non-confluent U87 cells were used for immunoprecipitation with IgG or anti-CD146 antibody. Western-blotting was then carried out using antibodies directed against VEGFR2, CD146 and integrin subunits αv and β3. Membrane receptor EPcam was used as a negative control **(A)**. U87 cells transfected or not with siRNA targeting β3 integrin were stimulated for 15 min with sCD146 or VEGF, and CD146 was immunoprecipitated before western blotting was done using antibody directed against total phospho-tyrosine **(B)**. Control, CD146-KO, VEGFR2-KO or β3-KO U87 cells were stimulated for 15 min with either sCD146 or VEGF. Lysates were then subjected to western-blotting and membranes were probed with either anti-phospho β3 Y759 **(C)** or anti-phospho VEGFR2 Y1054 **(D)**.

Average of 3 experiments is shown; *P<0.05, **P<0.01, ***p<0.001, experimental vs control.

**
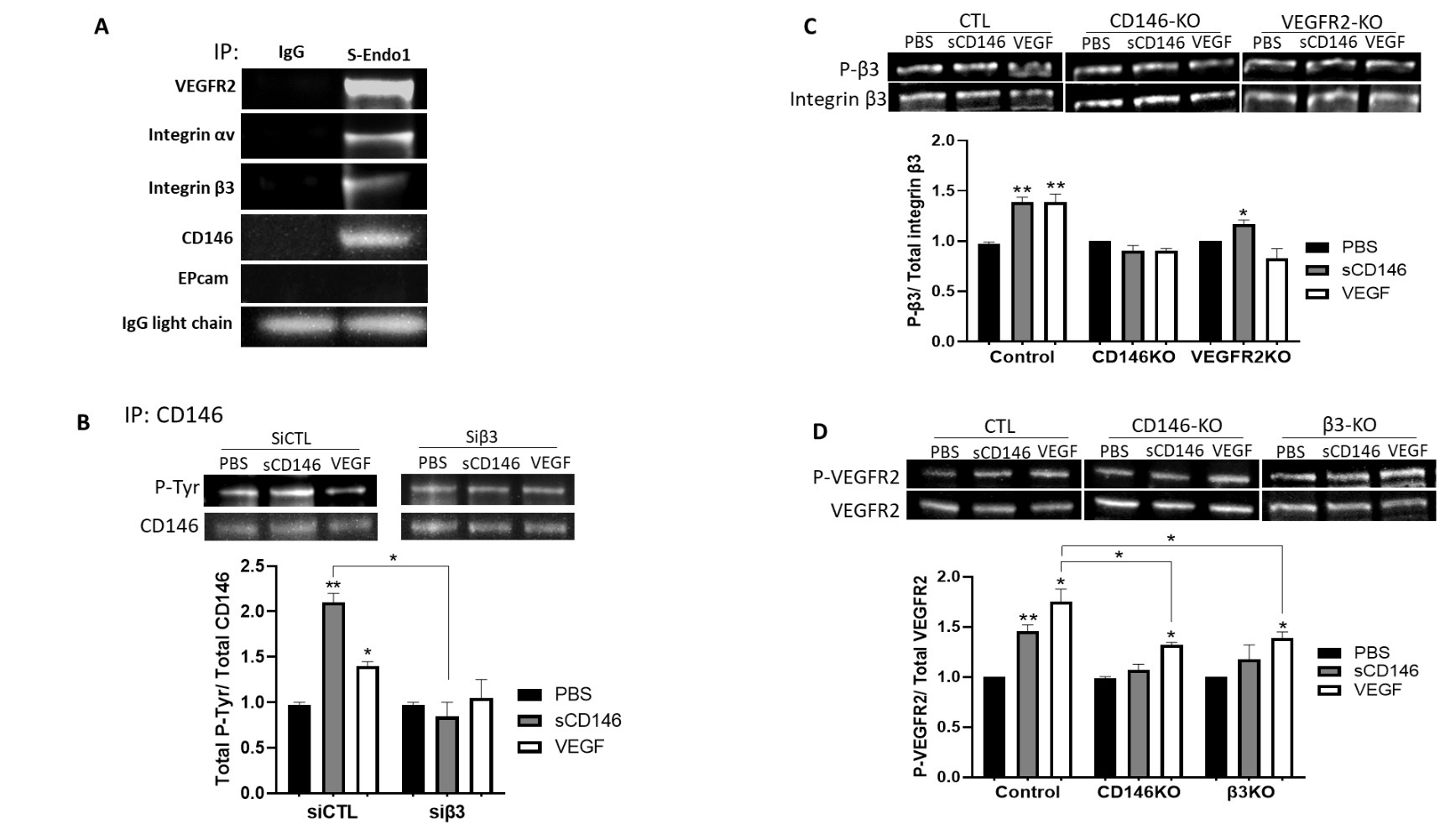
**

**Figure S12: sCD146 mediates its effects on U373 cells through a signalosome containing CD146, αvβ3, and VEGFR2.**

Non-confluent U373 cells were used for immunoprecipitation with IgG or anti CD146 antibody. Western-blotting was then carried out using antibodies directed against VEGFR2, CD146 and integrin subunits αv and β3. Membrane receptor EPcam was used as a negative control **(A)**. U373 cells transfected or not with siRNA targeting β3 integrin were stimulated for 25 min with sCD146 or VEGF, and CD146 was immunoprecipitated before western blotting was done using antibody directed against total phospho-tyrosine **(B)**. Control, CD146-KO, VEGFR2-KO or β3-KO U87 cells were stimulated for 25 min with either sCD146 or VEGF. Lysates were then subjected to western-blotting and membranes were probed with either anti-phospho β3 Y759 **(C)** or anti-phospho VEGFR2 Y1054 **(D)**.

Average of 3 experiments is shown; *P<0.05, **P<0.01, ***p<0.001, experimental vs control.

**
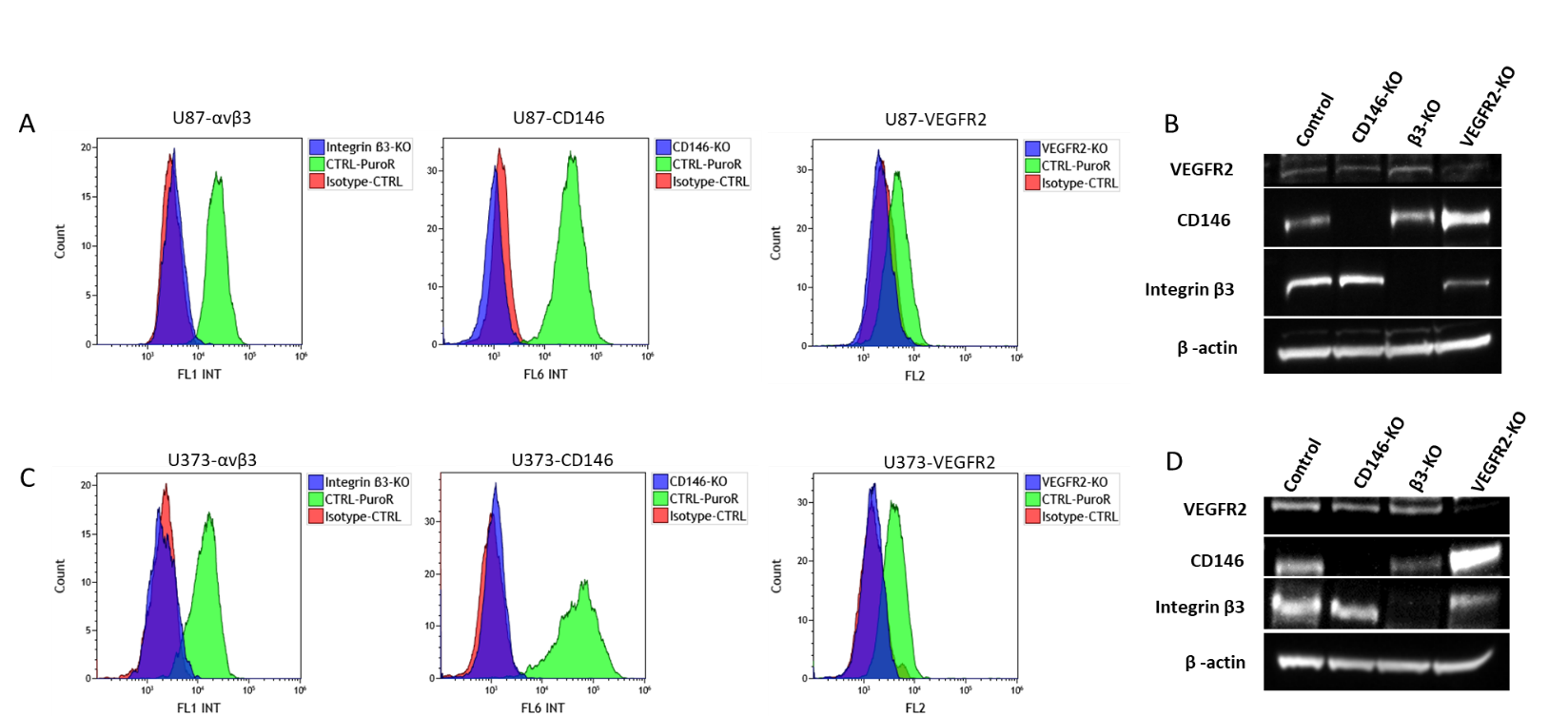
**

**Figure S13: Validating gene knock out in U87 and U373 cells.** Crispr/ Cas9 was used to knock out integrin β3 subunit, CD146, and VEGFR2 on U87 and U373 cell lines. Surface expression of the three proteins on U87 **(A)** and U373 **(C)** was assessed by flow cytometry. Immunoblots using anti-VEGFR2, anti-CD146, and anti- β3 antibodies on U87 **(B)** or U373 cell lysates are shown **(D)**.

**
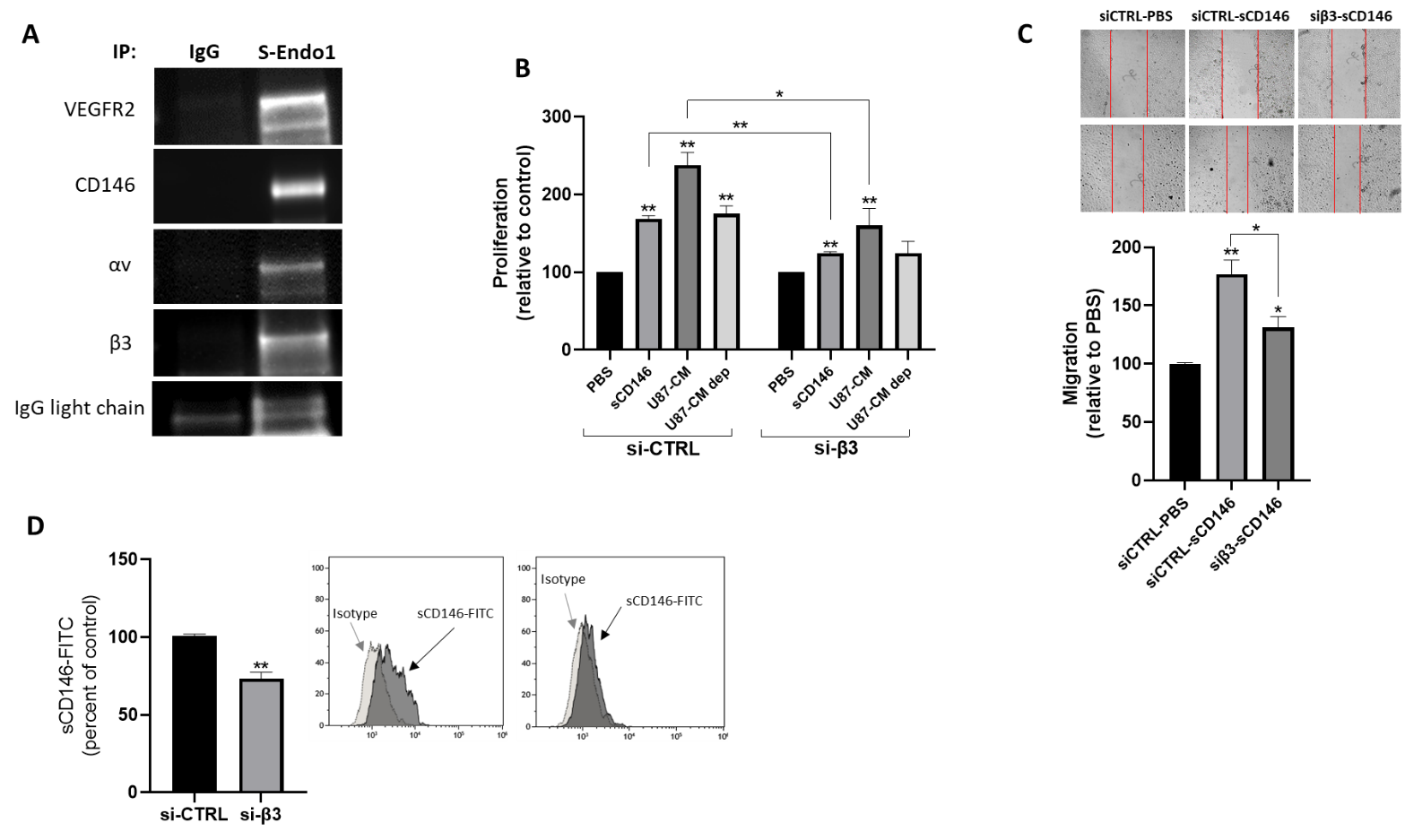
**

**Figure S14: Integrin αvβ3 associates with membrane CD146 and VEGFR2 on HUVECs and binds sCD146.** HUVECs were used for immunoprecipitation with IgG or anti-CD146 antibody. Western-blotting was then carried out using antibodies directed against VEGFR2, CD146 and integrin subunits αv and β3 **(A)**. HUVECs transfected or not with siRNA targeting β3 integrin were stimulated for 24 h with sCD146, conditioned media from U87 cells in EBM-2 (U87-CM), or U87-CM immunodepleted from sCD146 and effect on cell proliferation **(B)** and migration **(C)** was determined. HUVECs were transfected with siRNA targeting β3 integrin and sCD146-FITC binding was determined by flow cytometry **(D)**.

Average of 3 experiments is shown; *P<0.05, **P<0.01, experimental vs control.

**
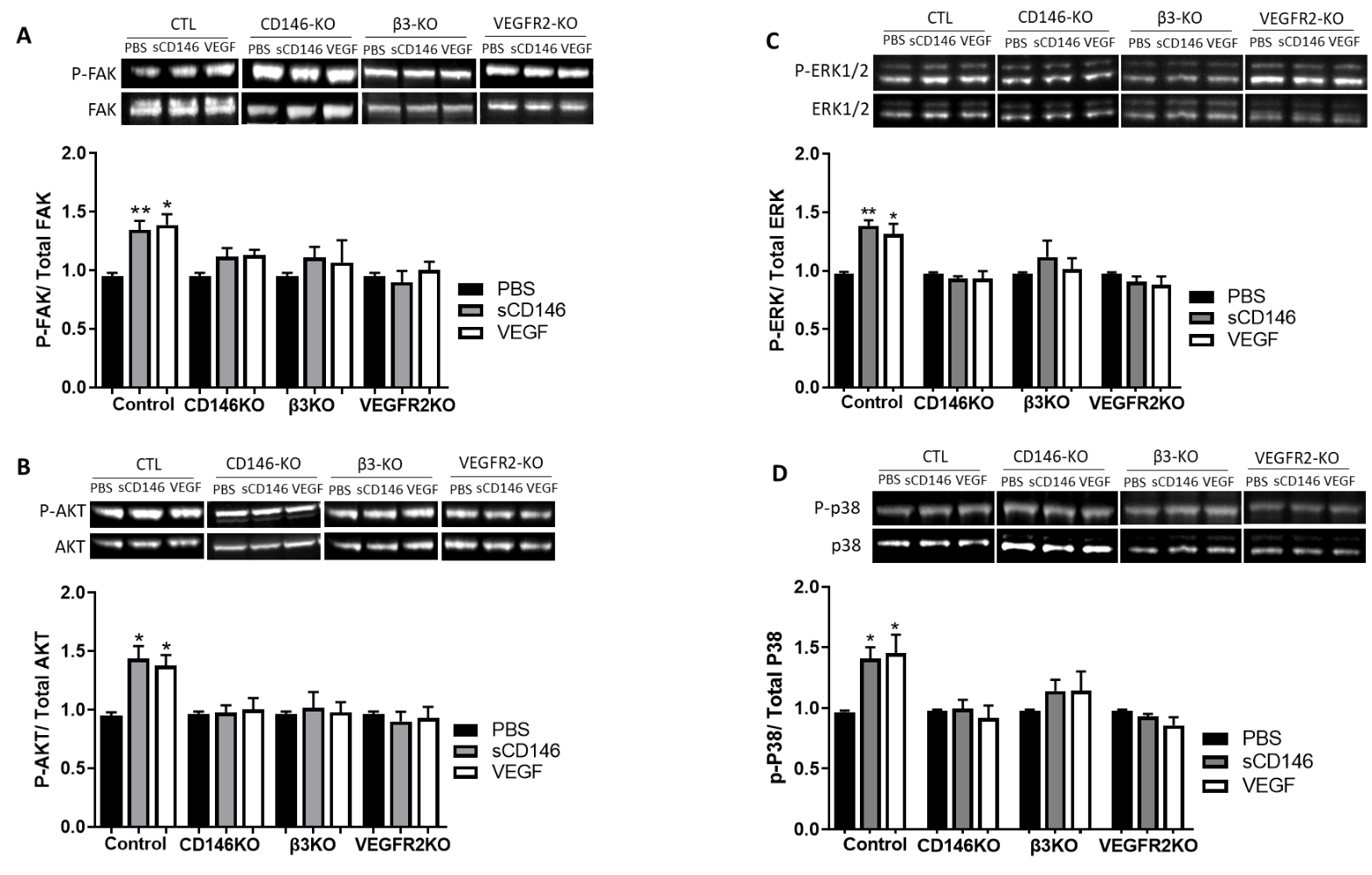
**

**Figure S15: CD146/VEGFR2/integrin αvβ3 signalosome mediates sCD146 and VEGF effects in U87 cells and activates common signaling pathways.**

U87 cells or knock-out cells were stimulated for 15 min with either sCD146 or VEGF and western-blotting was performed using p-FAK **(A)**, p-AKT **(B)**, p-ERK **(C)**, and p-P38 **(D)** antibodies. Normalization of phosphoproteins was done to the total protein.

Average of 3 experiments is shown; *P<0.05, **P<0.01, experimental vs control.

**
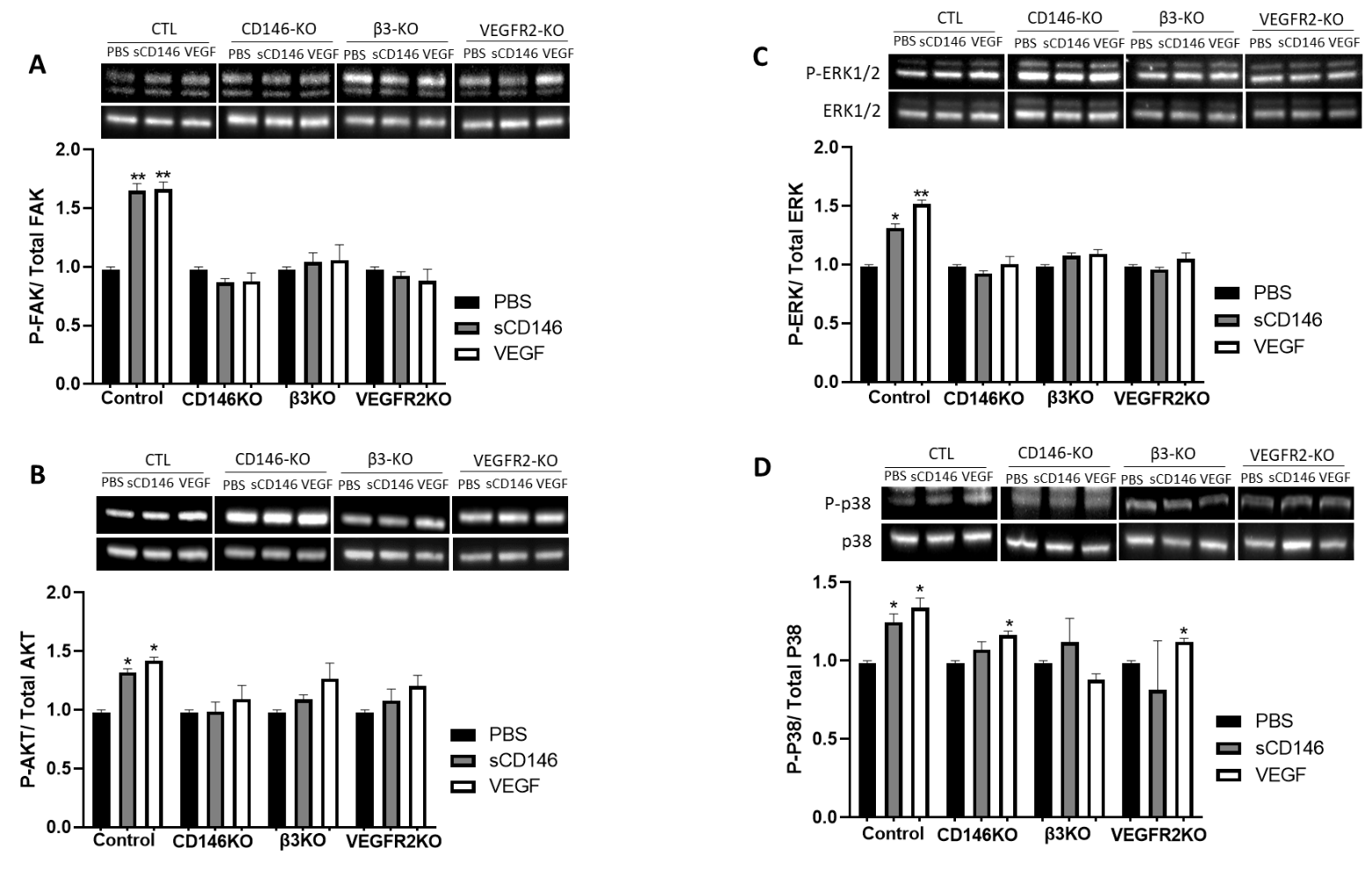
**

**Figure S16: CD146/VEGFR2/integrin αvβ3 signalosome mediates sCD146 and VEGF effects in U373 cells and activates common signaling pathways.**

U373 cells or knock-out cells were stimulated for 25 min with either sCD146 or VEGF and western- blotting was performed using p-FAK **(A)**, p-AKT **(B)**, p-ERK **(C)**, and p-P38 **(D)** antibodies. Normalization of phosphoproteins was done to the total protein.

Average of 3 experiments is shown; *P<0.05, **P<0.01, experimental vs control.

**
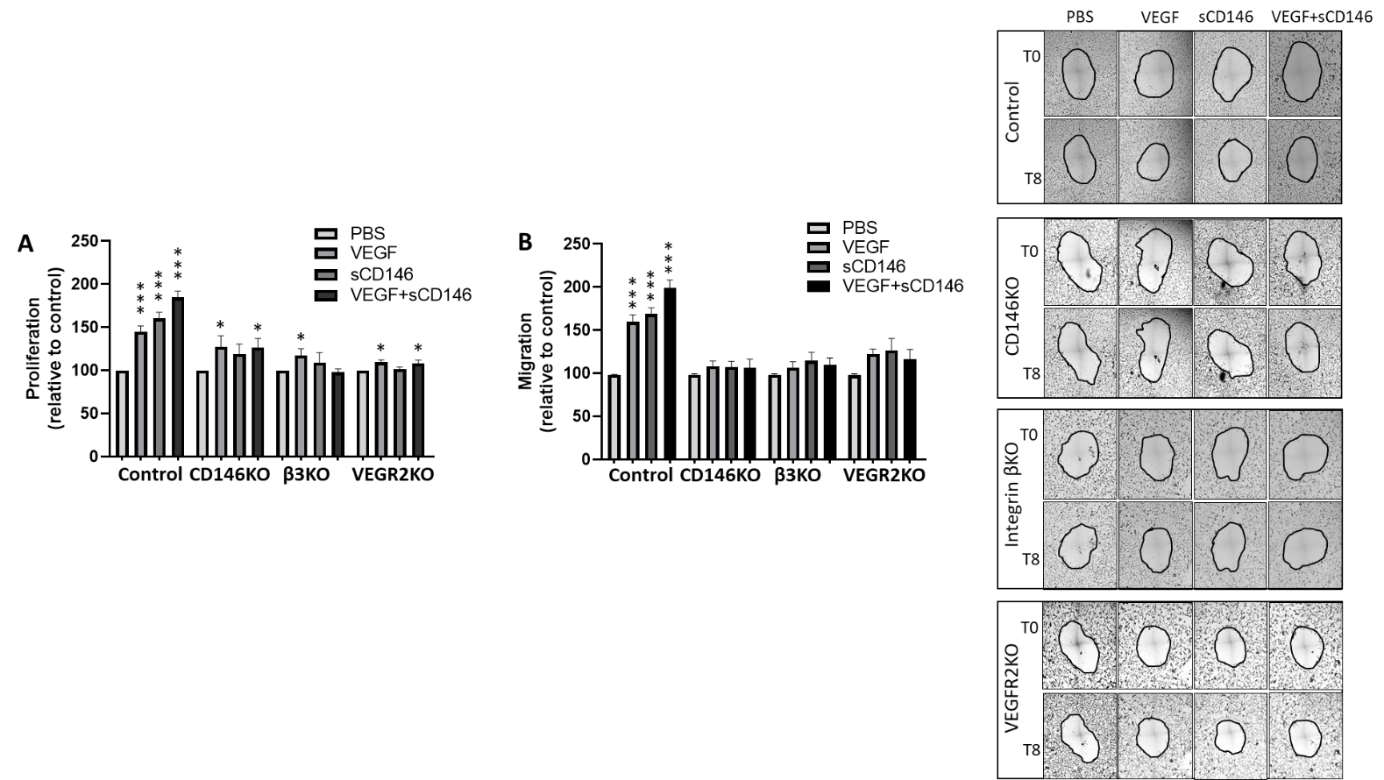
**

**Figure S17: CD146, integrin αvβ3, and VEGFR2 are essential for mediating sCD146 and VEGF effects on U87 cells.** U87 cells or knock-out cells were stimulated with sCD146 and /or VEGF and effect on cell proliferation **(A)** and migration **(B)** was evaluated. Representative images from 3-4 experiments are shown. *P<0.05, ***p<0.001, experimental vs control.

**
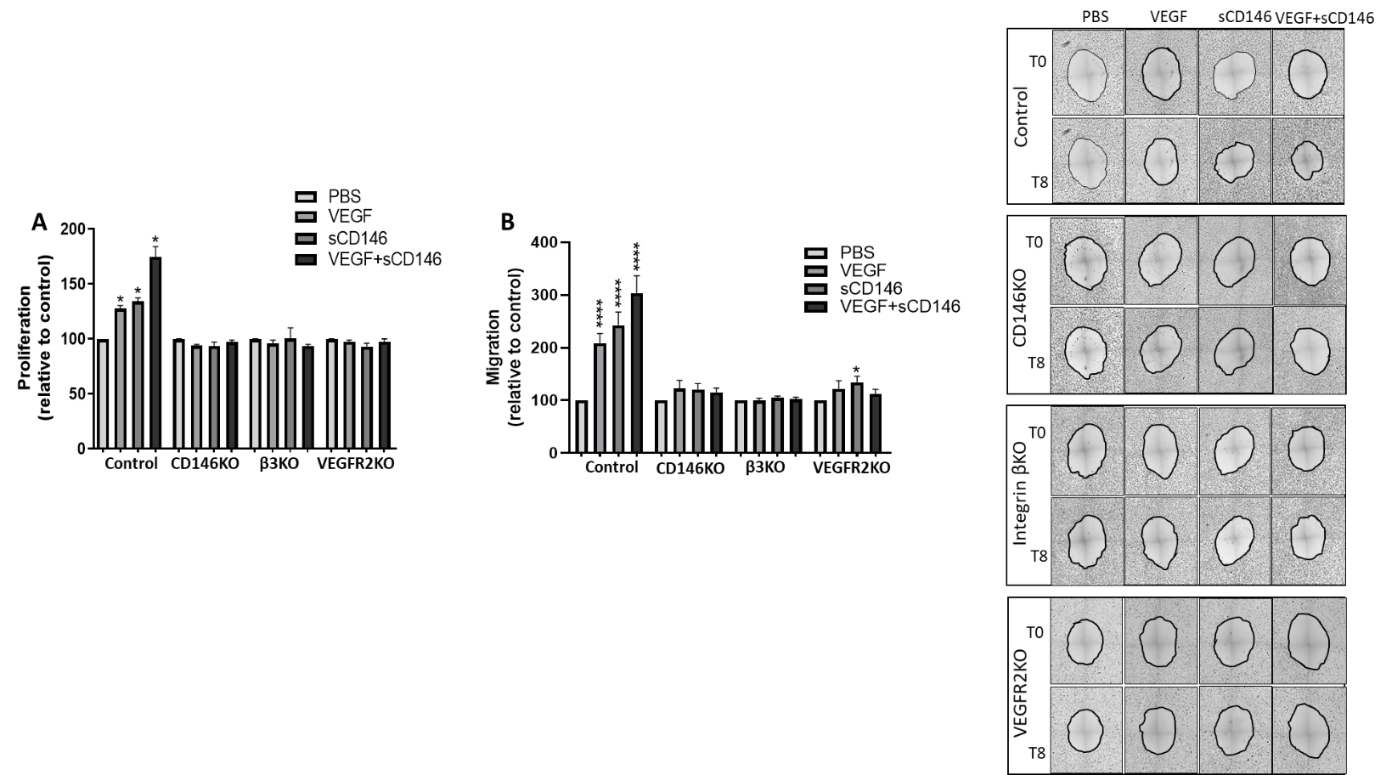
**

**Figure S18: CD146, integrin αvβ3, and VEGFR2 are essential for mediating sCD146 and VEGF effects on U373 cells.** U373 cells or knock-out cells were stimulated with sCD146 and /or VEGF and effect on cell proliferation **(A)** and migration **(B)** was evaluated. Representative images from 3-4 experiments are shown. *P<0.05, ***p<0.001, experimental vs control.

**
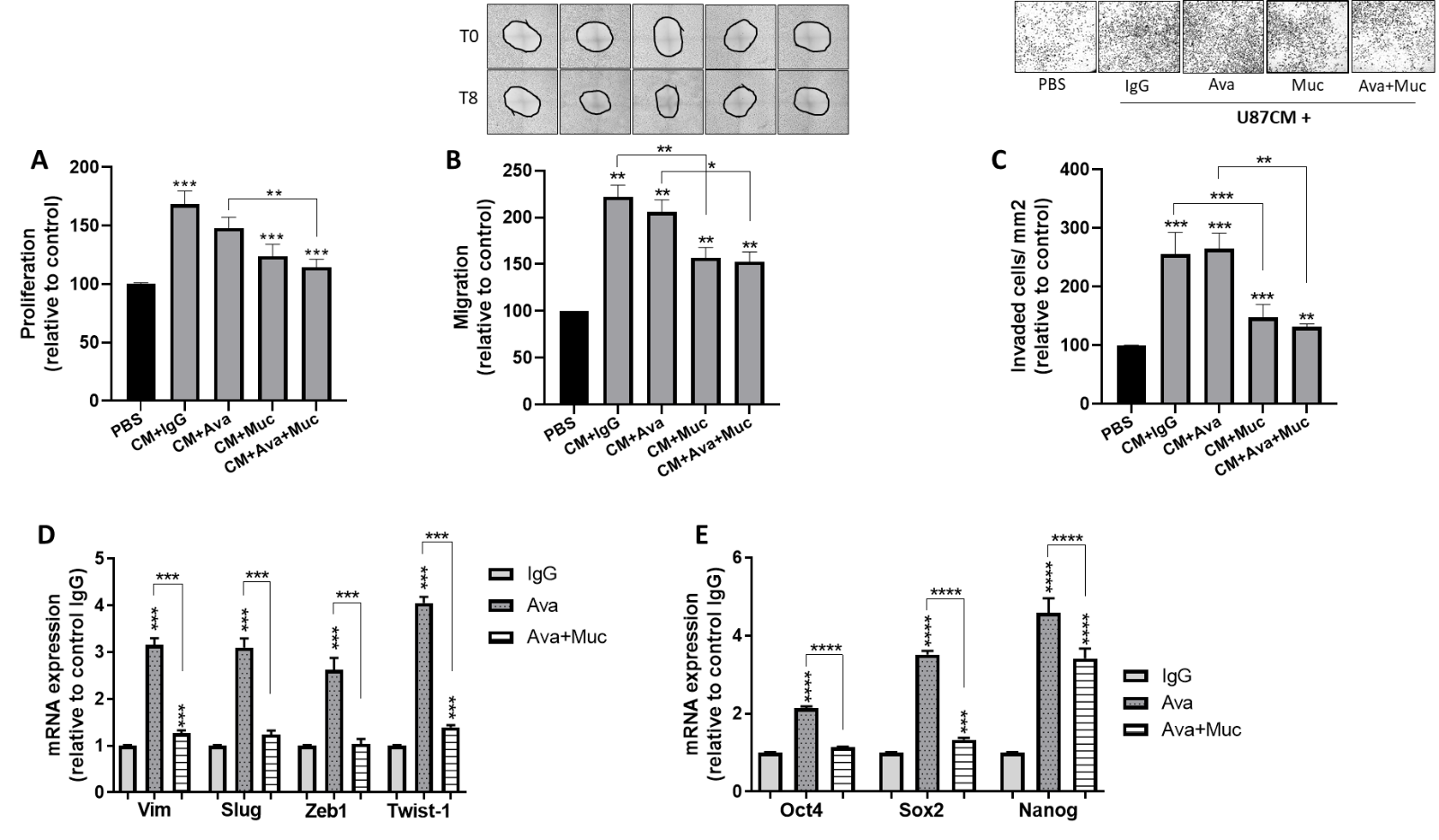
**

**Figure S19: Humanized anti-sCD146 antibody mucizumab significantly decreases U373 cell proliferation, migration and invasion and hampers CSC and EMT in-vitro.**

U373 cells were primed with conditioned media (CM) pre-treated in the presence of irrelevant IgG, bevacizumab (Avastin), Mucizumab, or combination of both antibodies for proliferation **(A)**, migration **(B)**, and invasion assays **(C)**. EMT and CSC markers were also examined at the mRNA (**D and E**) level.

Average of 3 experiments is shown; *P<0.05, **P<0.01, ***p<0.001, experimental vs control.

**
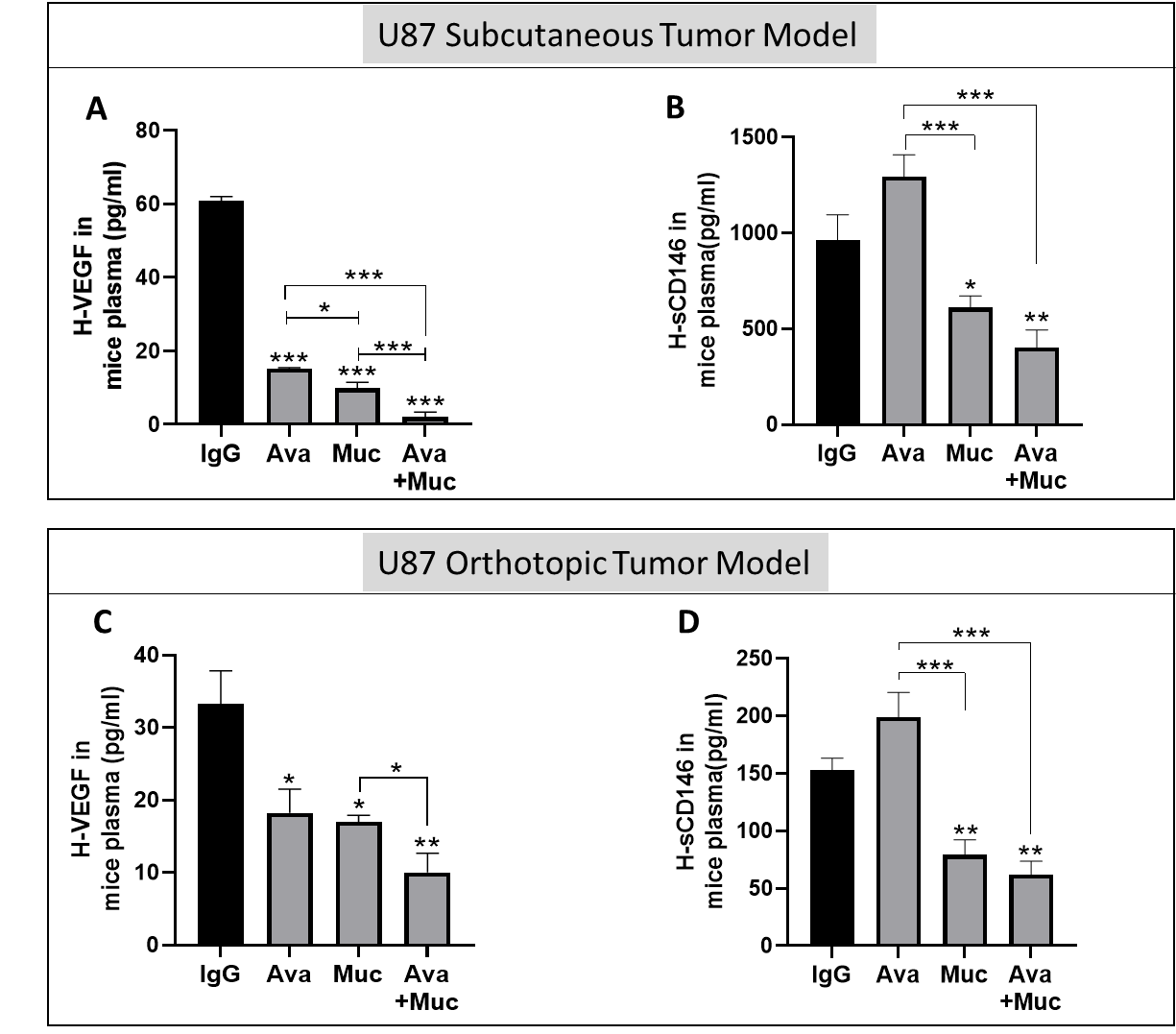
**

**Figure S20: Humanized anti-sCD146 mucizumab significantly decreases human sCD146 and human VEGF in two different mouse models of glioblastoma.**

Nude mice were either subcutaneously or orthotopically injected with U87 cells and treated with IgG, bevacizumab (Avastin), mucizumab or Avastin+mucizumab. Quantification of human VEGF **(A, C)** and sCD146 **(B, D)** in the sera of treated mice was performed. 5 mice were used in each group.

*P<0.05, **P<0.01, ***p<0.001, experimental vs control.

**
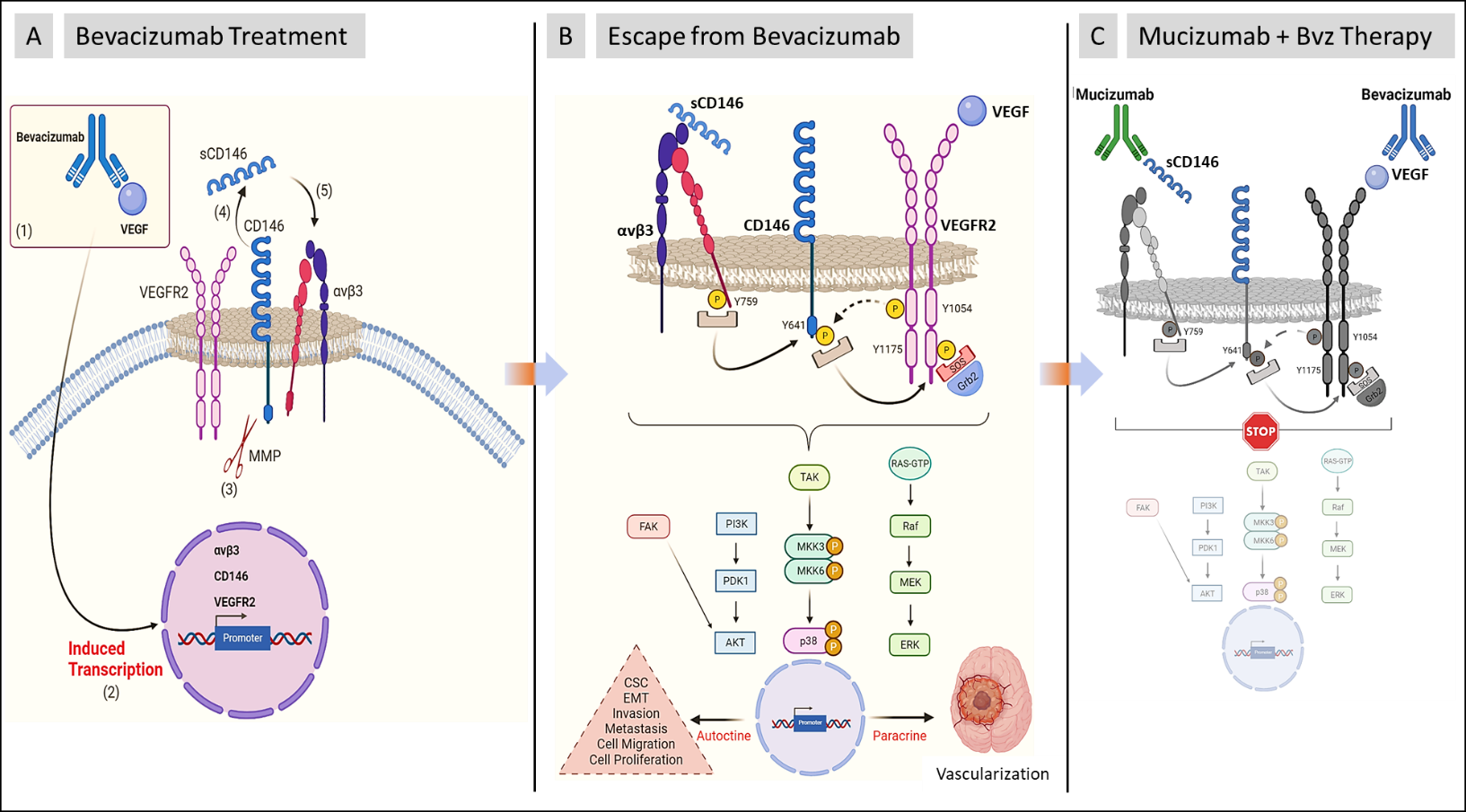
**

**Figure S21: Illustrative summary describing the mechanism of resistance to bevacizumab in CD146-positive glioblastoma cells.**

CD146-positive glioblastoma cells respond to bevacizumab-induced VEGF deficiency by upregulating integrin αvβ3, CD146, and VEGFR2 expression. Membrane CD146 is then shed by MMPs to produce sCD146. sCD146, in turns, binds integrin αvβ3 and induces β3 subunit phosphorylation. Phosphorylated β3 recruit adaptor proteins and kinases which phosphorylates CD146 at Y641. In turn, phosphorylated CD146 changes conformation and interacts with cytosolic adaptor proteins and kinases which permits to activate and phosphorylate VEGFR2 even in the absence of VEGF. Consequently, multiple signaling pathways are initiated including P38 MAPK, ERK 1/2, AKT, and FAK pathways which induce cells’ proliferation, migration, invasion, mesenchymal transition, and cancer stem cell generation. Also, via paracrine signaling on endothelial cells, sCD146 binds integrin αvβ3 and induces cells proliferation that subsequently enhances tumor vascularization.
